# Noninvasive monitoring of vascular alterations in mice with acute lower limb ischemia using multimodal photoacoustic imaging

**DOI:** 10.1002/btm2.70005

**Published:** 2025-02-17

**Authors:** Heng Wang, Keyi Fan, Yijie Ning, Chuanlong Lu, Yuhang Zhang, Qian Wang, Hongjiu Zhang, Yaling Li, Zeyu Zhang, Xiaohua Jia, Sheng Yan, Ruijing Zhang, Honglin Dong

**Affiliations:** ^1^ Department of Vascular Surgery The Second Hospital of Shanxi Medical University Taiyuan People's Republic of China; ^2^ Vascular Institute of Shanxi Medical University Shanxi Medical University Taiyuan People's Republic of China; ^3^ Department of Ultrasound The Second Hospital of Shanxi Medical University Taiyuan People's Republic of China; ^4^ Key Laboratory of Molecular Imaging of Chinese Academy of Sciences, Institute of Automation Chinese Academy of Sciences Beijing People's Republic of China; ^5^ Department of Ultrasound Shuozhou Grand Hospital of Shanxi Medical University Shuozhou People's Republic of China; ^6^ Department of Nephrology The Second Hospital of Shanxi Medical University Taiyuan People's Republic of China

**Keywords:** acute lower limb ischemia, continuous monitoring, duplex ultrasound, near‐infrared II imaging, optical coherence tomography angiography, real‐time and wide‐field laser speckle imaging

## Abstract

Acute limb ischemia (ALI), especially acute lower limb ischemia (ALLI), is a common clinical vascular emergency with high amputation and mortality rates. However, major challenges exist in rapidly diagnosing and assessing collateral vascular compensatory capacity, leading to appropriate clinical treatment strategies to improve limb preservation and reduce recurrence rates. Traditional imaging methods, such as digital subtraction angiography (DSA) and ultrasound, have high demands on patient kidney function and operator maneuvers and are unable to monitor the temporal and spatial variability of collateral circulation establishment in the limb. In this study, we report the first combined use of real‐time and wide‐field laser speckle imaging (RFLSI), near‐infrared two‐zone imaging (NIR‐II), duplex ultrasound (DUS), and optical coherence tomography angiography (OCTA) for the diagnosis and monitoring of ALLI in male C57 and ICR mice. The RFLSI assesses overall perfusion, the NIR‐II allows rapid diagnosis and monitoring of the establishment of collateral circulation, DUS monitors muscle tenderness and stiffness, and OCTA detects microcirculation in the skin of the limb, which is confirmed by histopathological testing. Overall, the results of this study demonstrate the ability to accurately diagnose and detect ALLI with the help of a variety of optical and acoustic imaging devices, with significant clinical translational implications for intervention in clinical decision‐making preoperatively and prognostic assessment postoperatively.

**Translational Impact Statement**

This study demonstrates that the combined use of four optical and acoustic imaging devices, RFLSI, NIR‐II, DUS, and OCTA, allows for the rapid diagnosis of ALLI the monitoring of skin microcirculation and muscle activity, and the establishment of collateral circulation. This finding holds great promise for future intervention in clinical decision making and assessment of prognosis.

## 1 INTRODUCTION

Acute limb ischemia (ALI) is defined as a sudden decrease in arterial perfusion to a limb that potentially threatens limb survival and has a duration of symptoms of ≤2 weeks.^1^, ^2^ Of these, acute lower limb ischemia (ALLI) is more common than acute upper limb ischemia in clinical practice.^3^ The most common causes of ALLI are arterial embolism, autoarterial thrombosis, peripheral aneurysm, entrapment, and traumatic arterial injury. Historical data from Sweden and the United Kingdom suggest an incidence rate of 3–14 per 100,000 person‐years, with the vast majority aged >80 years.^4^, ^5^ A study from the United States revealed that patients with ALLI had a mortality rate of 9.0%, an amputation rate of 6.4%, and a one‐year mortality rate of 42.5%.^6^ Therefore, ALLI, as a vascular emergency, requires rapid diagnosis, assessment of limb viability, and implementation of treatment to maximize the likelihood of limb preservation. However, in clinical practice, the classic “six syndromes” (pain, pallor, pulselessness, poikilothermia, paresthesia, and paralysis) are not typical in patients.^7^ Since skeletal muscle tolerates ischemia for 4–6 h, the longer a patient is symptomatic, the less likely he or she is to preserve the limb, and the higher the rate of recurrence and mortality.^1^, ^8^ Therefore, the ability to make a timely diagnosis, assess limb survival, and administer appropriate treatment becomes critical.

Currently, digital subtraction angiography (DSA) is considered the gold standard for the diagnosis of ALI.^1^, ^9^ DSA can provide an etiologic reference and treatment. However, it is invasive and not suitable for patients with severe renal insufficiency.^10^ In addition, decision‐making on revascularization strategies for these patients can be improved by rapidly obtaining noninvasive imaging, such as duplex ultrasound (DUS), computed tomography angiography (CTA), and magnetic resonance angiography (MRA), to determine anatomic structures as well as the etiology of ALLI.^11^, ^12^, ^13^ These imaging methods still face many dilemmas, such as limited clinical applicability in acute situations, insufficient visualization of the image, potential for iatrogenic acute kidney injury, slow examination time, and high price. Therefore, imaging techniques that allow rapid detection, visualization, easy operation, and dynamic real‐time observation are particularly important.

In vivo imaging is an important tool for studying abnormal pathologic processes in disease. Laser speckle imaging (LSI) has been used to assess microcirculation in ophthalmology and hemodynamics in the foot, and is an imaging tool that allows the quantitative assessment of blood flow.^14^, ^15^, ^16^ LSI offers the advantages of short measurement times, no contact and continuous inspection. Compared with near‐infrared (NIR) I imaging (650–900 nm), near‐infrared II fluorescence imaging (1000–1700 nm) has the advantages of deeper tissue penetration, higher spatial resolution, and reduced normal tissue scattering.^17^ Indocyanine green (ICG) is an FDA‐approved NIR agent widely used for liver fibrosis diagnosis, tumor resection guidance, and identification of sentinel lymph nodes, and has great promise for vascular imaging.^18^, ^19^, ^20^ Optical coherence tomography (OCT) has become a powerful noninvasive tool used for retinal studies because it allows detailed visualization of retinal morphology and the vascular system at near cellular resolution.^21^ Optical coherence tomography angiography (OCTA) is a functional extension of OCT that allows label‐free visualization of microvessels.^22^

**FIGURE 1:**
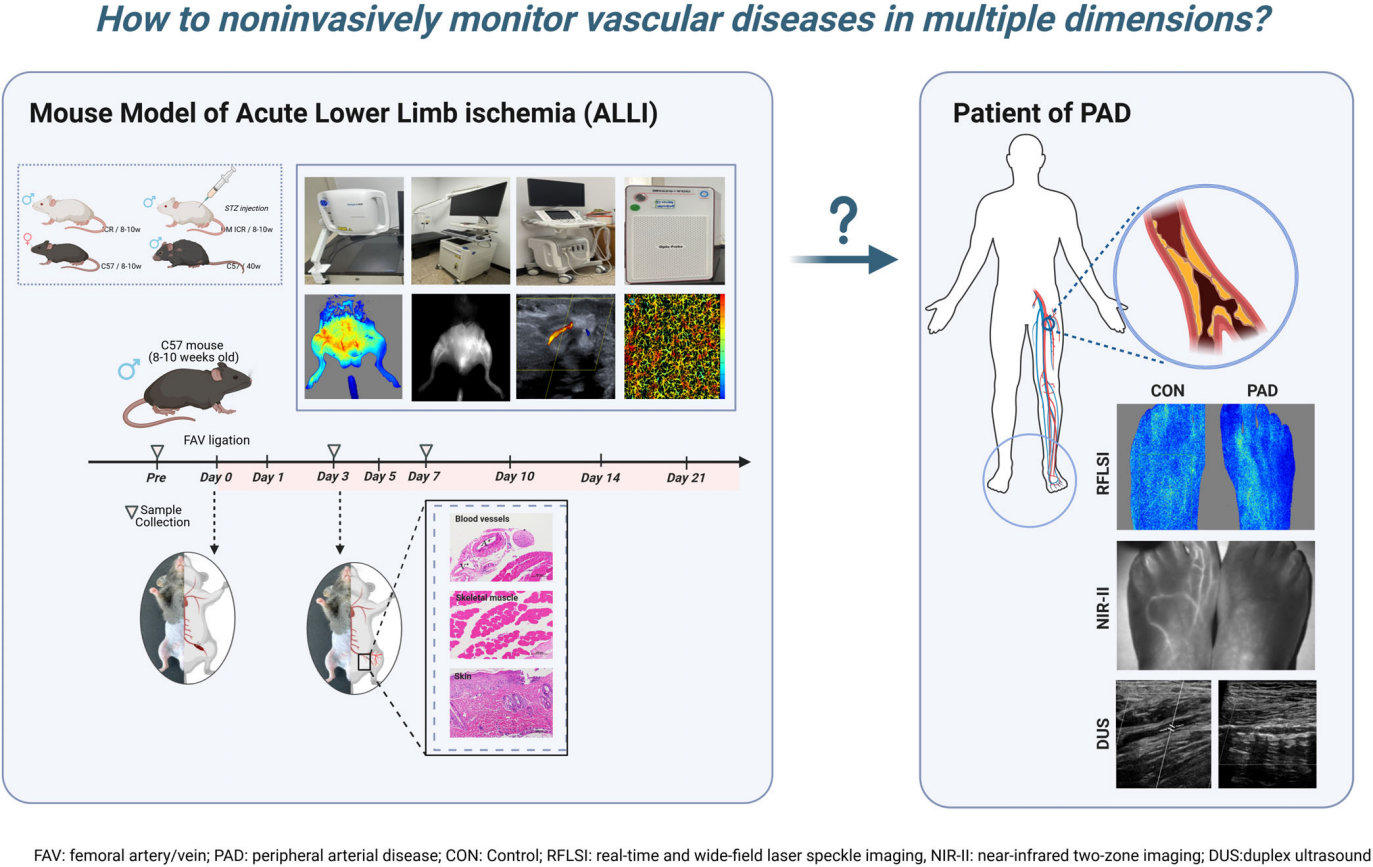
Schematic diagram of the diagnosis and monitoring of ALLI based on the RFLSI, NIR‐II, ultrasound, and OCTA imaging.

In this study, we utilized four optical and acoustic imaging devices for the diagnosis, monitoring, and prognostic evaluation of ALLI (Figure 1). Disease progression in ALLI patients was visualized and quantified by noninvasive imaging and validated with histopathology. This study utilized preclinical imaging to complement the clinical imaging of ALLI and provide a more comprehensive method of perfusion monitoring that will serve to guide clinical decision‐making and assess patient prognosis. This study offers hope for future clinical translation.

## 2 RESULTS

### 2.1 Ultrasound results and pathological features for evaluating model development

After the ALLI model was established, ultrasonic results and pathological H&E staining of the mice 3 and 7 days after ALLI and the control group were selected to verify the accuracy of the model establishment. The DUS results revealed that femoral artery (FA) patency was significantly impaired and that a small amount of collateral circulation was established 3 days after modeling compared with that in the control group (Figure S1a). After 7 days of modeling, FA patency increased significantly, and more collateral circulation was established (Figure S1a).

**FIGURE 2:**
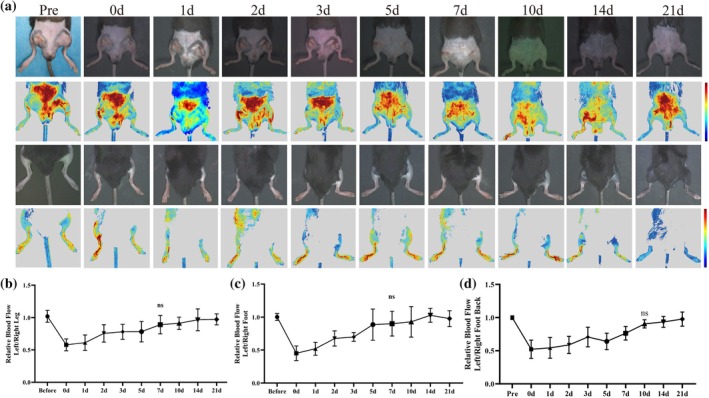
RFLSI imaging for the diagnosis and continuous monitoring of ALLI (male C57) (*n* = 5). (a) ALLI mice were continuously monitored via RFLSI imaging and photographs were taken at the following times: Before (pre), the operation (0d); and at 1, 2, 3, 5, 7, 10, 14, and 21 days after operation. Quantitative values of relative blood flow (b) in the damaged legs, (c) in the damaged feet, and (d) in the dorsal feet of ALLI mice (*n* = 5).

The femoral arteriovenous veins of the lower limbs of the mice were removed and stained (Figure S1b). Three days after ALLI, the mice had thrombi in the proximal FA but not in the vein; no thrombi were present in the distal FA. Seven days after ALLI, there was a thrombus in the femoral vein but not in the artery proximal to the heart; there was no thrombus in the FA proximal to the heart. Next, mouse leg muscles were sampled and stained with H&E (Figure 2c). Skeletal muscle cells in the normal group were obtuse, polygonal, structurally intact, and uniform in size. ALLI 3d mice had collapsed and lysed skeletal muscle cells that were atrophied and deformed, with infiltration of inflammatory cells and even hyperplasia of fibrous tissue (Figure S2). In contrast, the skeletal muscle of the ALLI 7d group was significantly improved.

The above results confirmed that the ALLI model was successfully constructed from both DUS and pathological perspectives.

### 2.2 RFLSI imaging assesses the overall blood flow of the lower limbs

First, the ALLI model was constructed by FA ligation on the left lower limb of mice (male C57‐FA ligation, male C57‐artery and vein [FAV] ligation, female C57, T2D, ICR, and elderly mice). The ALLI model (male C57) was continuously monitored for 3 weeks. Blood perfusion to the left lower extremity was lowest on the postoperative day, and blood flow did not significantly differ from that of the controls on the seventh day (Figure 2a–d). Interestingly, the affected limbs of ALLI model mice presented significant contractures, skin breakdown, increased skeletal muscle stiffness, and lameness in the first 7 days (Figure 2a). Starting from Day 7, the mice gradually recovered the function of the affected limb. Blood perfusion recovery was evaluated by the ratio of the signal value of the ligated side to that of the contralateral side (Figure S3). All the quantified results revealed a gradual recovery of lower limb blood flow after surgery (Figure 2b–d). Detailed RFLSI images and mouse photos of male C57‐FA ligation (Figures S4 and S5), male C57‐FAV ligation (Figures S6 and S7), female C57 (Figure S8), T2D (Figure S9), ICR (Figures S10 and S11), and elderly (Figures S12 and S13) mice were added to the Supporting Information.

### 2.3 NIR‐II imaging visualizes the vascular morphology of the lower limb

**FIGURE 3:**
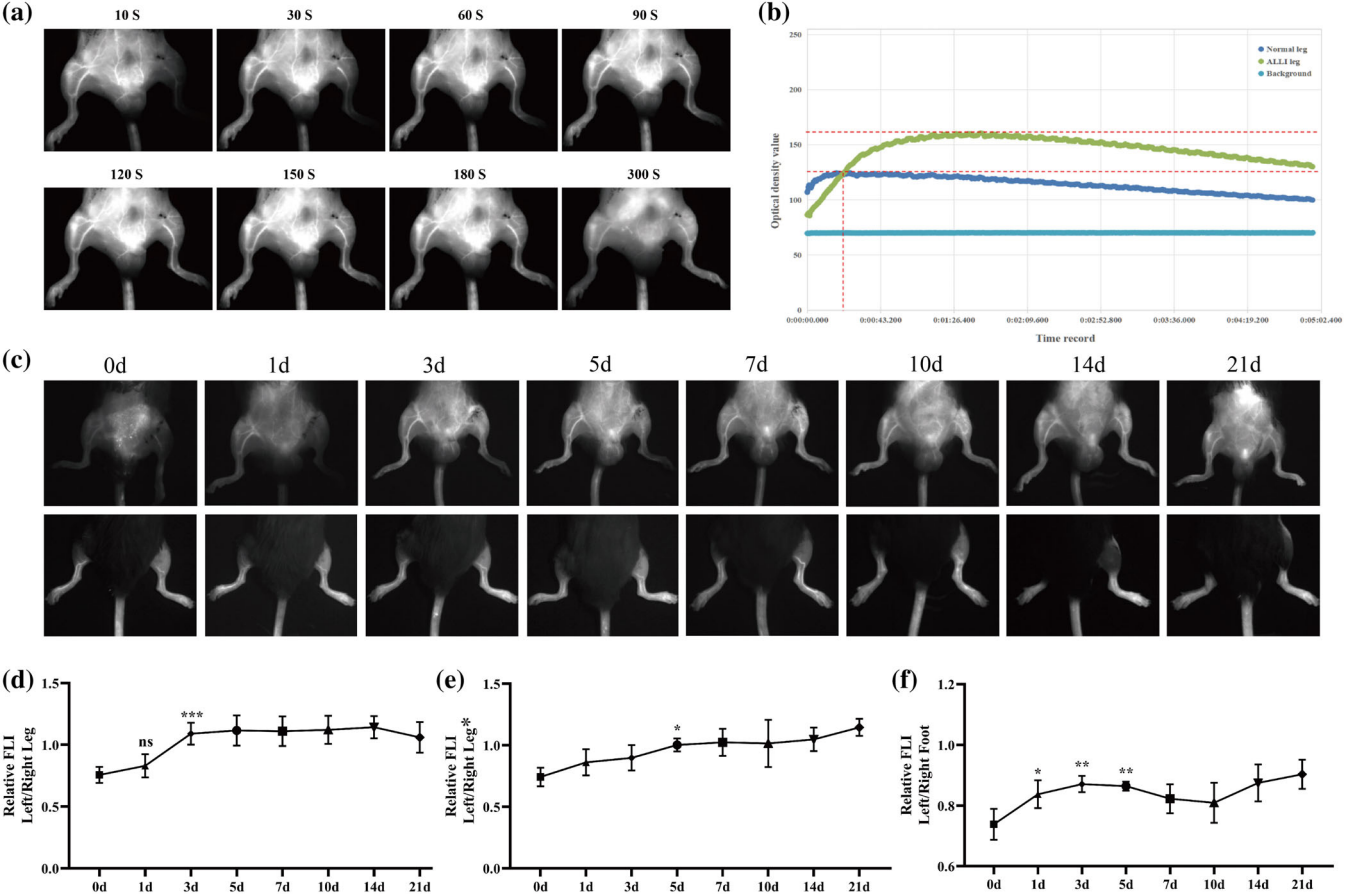
NIR‐II imaging for the diagnosis and continuous monitoring of ALLI (male C57) (*n* = 5). (a) NIR‐II imaging was performed on ALLI mice immediately after surgery, and the monitoring times were 10, 30, 60, 90, 120, 150, 180, and 300 s after ICG injection. (b) Time‐intensity curve of ICG signal intensity for NIR‐II imaging of ALLI mice immediately after surgery. (c) NIR‐II imaging was continuously monitored in ALLI mice at the following time points: Operation (0d), after 1, 3, 5, 7, 10, 14, and 21 days after the operation. NIR‐II imaging 10 s after ICG injection was selected as a representative image. (d) The ratio of fluorescence intensity of the left leg to that of the right leg, (e) fluorescence intensity of the left femoral artery to the right femoral artery, (f) fluorescence intensity of the left dorsal foot to that of the right dorsal foot. *p < 0.05, **p < 0.01, ***p < 0.001.

ICG was injected into ALLI model mice (male C57‐FA ligation, male C57‐FAV ligation, female C57, T2D, ICR, and elderly mice) via the tail vein for NIR‐II imaging. ALLI mice (male C57) were then continuously monitored, and the time was divided into three segments on the basis of their fluorescence signal characteristics and anatomy (Figure 3a,b). At 0–20 s, the fluorescence signal of the affected limb gradually increased but was lower than that of the contralateral side because of the ligation of the FA in the left leg. At 20–90 s, the fluorescence signal of the affected limb continued to rise to a peak due to poor blood return caused by FA ligation. At 90–300 s, the fluorescence signal in the affected limb began to diminish due to the presence of microcirculation, but was always greater than the contralateral fluorescence signal. In addition, ALLI model mice were continuously monitored for 21 days, and fluorescence signal maps were collected 10 s after ICG injection (Figure 3c). On Day 7, the femoral arteriovenous blood flow status of the affected side of the model mice returned to normal. As shown in Figure S14a, collateral circulation was found in the affected limb of the ALLI model mice, and these vessels were increased in size and significantly dilated. In addition, the fluorescence signal intensity and morphology at the FA of the left affected limb of ALLI mice on Day 7 were similar to those of the right side, but there was still a significant gap at the ligation point (Figure S14b). In this study, three areas were selected to quantify the NIR intensity (Figure S15), including the bilateral leg, bilateral points at the FA, and bilateral dorsal foot. The ratio of the fluorescence intensity of the left leg to that of the right leg increased significantly at 3 days and tended to be stable at 3–14 days (Figure 3d). The ratio decreased slightly at 21 days and was close to 1. The ratio of the fluorescence intensity of the left FA to that of the right FA gradually increased after surgery and was greater than 1 at 21 days, indicating that the left FA had greater fluorescence intensity (Figure 3e). The ratio of the fluorescence intensity of the left dorsal foot to that of the right dorsal foot was always less than 1, indicating that the perfusion recovery of the dorsal foot was worse than that of the leg (Figure 3f). Representative NIR‐II images of male C57‐FA ligation (Figure S16), male C57‐FAV (Figure S16), female C57 (Figure S16), T2D (Figure S17), ICR (Figure S16), and elderly (Figure S17) mice were added to the Supporting Information.

### 2.4 DUS imaging and staining analysis detect collateral circulation establishment and muscle elasticity

**FIGURE 4:**
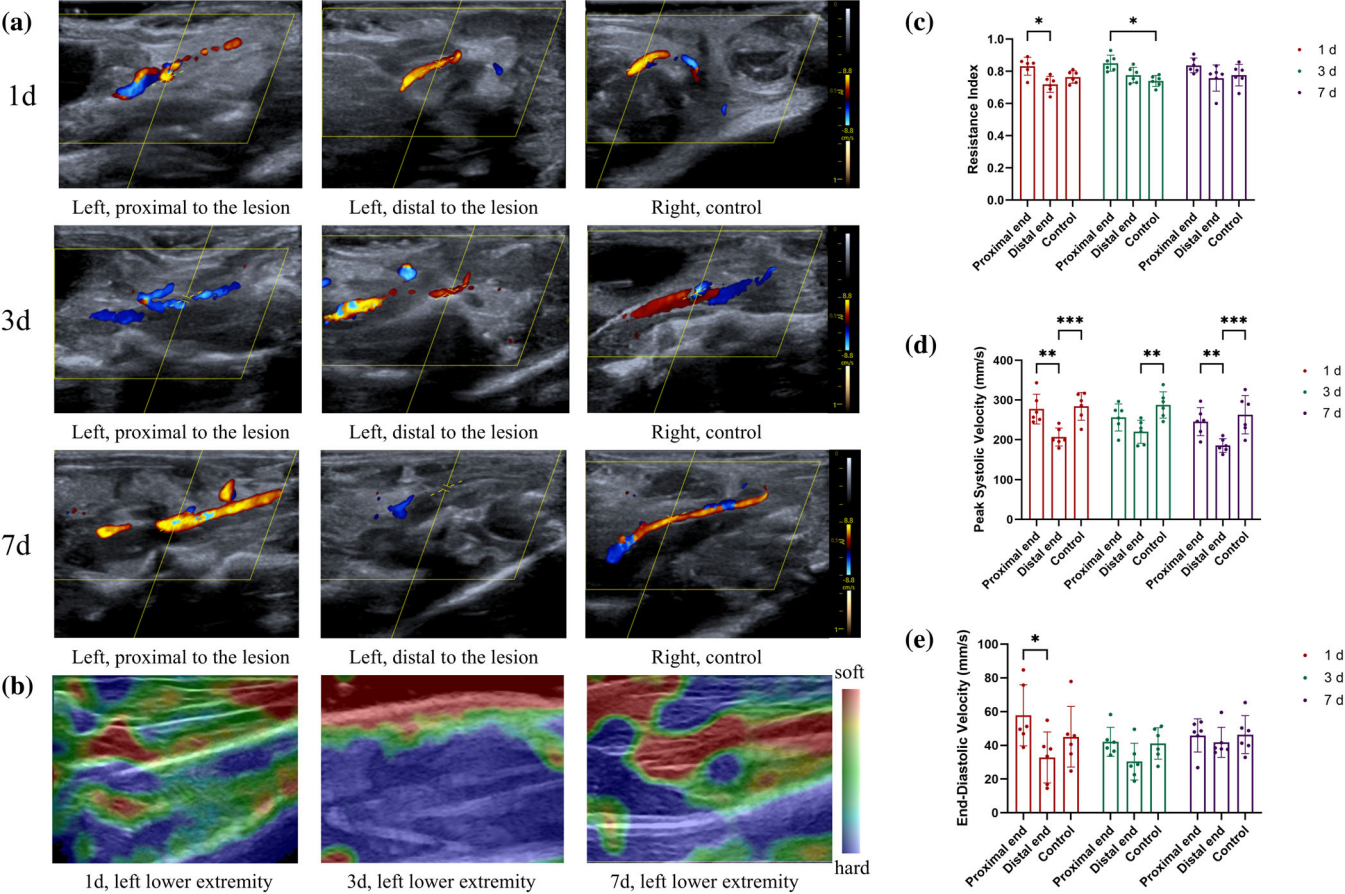
Ultrasound imaging for the diagnosis and continuous monitoring of ALLI (male C57) (*n* = 5). (a) Representative ultrasound images of ALLI mice at 1, 3, and 7 days after operation. (b) Ultrasound elastography of the legs of ALLI mice was continuously monitored at the following timepoints: 1, 3, and 7 days after the operation. (c–e) The resistance index (RI), peak systolic velocity (PSV) and end‐diastolic velocity (EDV) were used to quantify the DUS results of the ALLI mice 1, 3, and 7 days after the operation. *p < 0.05, **p < 0.01, ***p < 0.001.

DSA is currently the gold standard for the diagnosis of ALLI, whereas the diagnostic accuracy of DUS is not significantly different from that of DSA or CTA.^23^ Since the diameter of blood vessels in mice is much smaller than that in humans, the selection of a customized ultrasound probe and microfluidic acquisition pattern is critical. As shown in Figure 4a, control mice had intact popliteal and saphenous arteries, and broken ends of FA ligations were observed in the ALLI model mice, with no blood flow passing through their distal ends. Moreover, DUS imaging was performed at 1, 3, and 7 days after ALLI in male C57 mice (Figure 4a). DUS images of female C57, T2D, ICR, and elderly mice are shown in Figure S18. Muscle stiffness was assessed using ultrasound elastography (Figure 4b). At 1 day postsurgery, the elastic coefficient of the gastrocnemius muscle of the affected limb decreased significantly in ALLI mice, but the elastic coefficient gradually recovered at 3 and 7 days, indicating the recovery of muscle stiffness (Figure S19). The resistance index (RI), peak systolic velocity (PSV), and end‐diastolic velocity (EDV) were used to quantify the DUS results. For the RI, the distal end of the ligation site was greater than the proximal end 1 day after surgery, and the distal end was greater than the control at 3 days after surgery (Figure 4c). For the PSV, the distal PSV was lowest at 1, 3, and 7 days after surgery (Figure 4d). For the EDV, the distal EDV was lower than the proximal EDV at 1 day after surgery (Figure 4e). These parameters reflect the hemodynamic changes associated with ALLI and contribute to the refined management of the disease.

**FIGURE 5:**
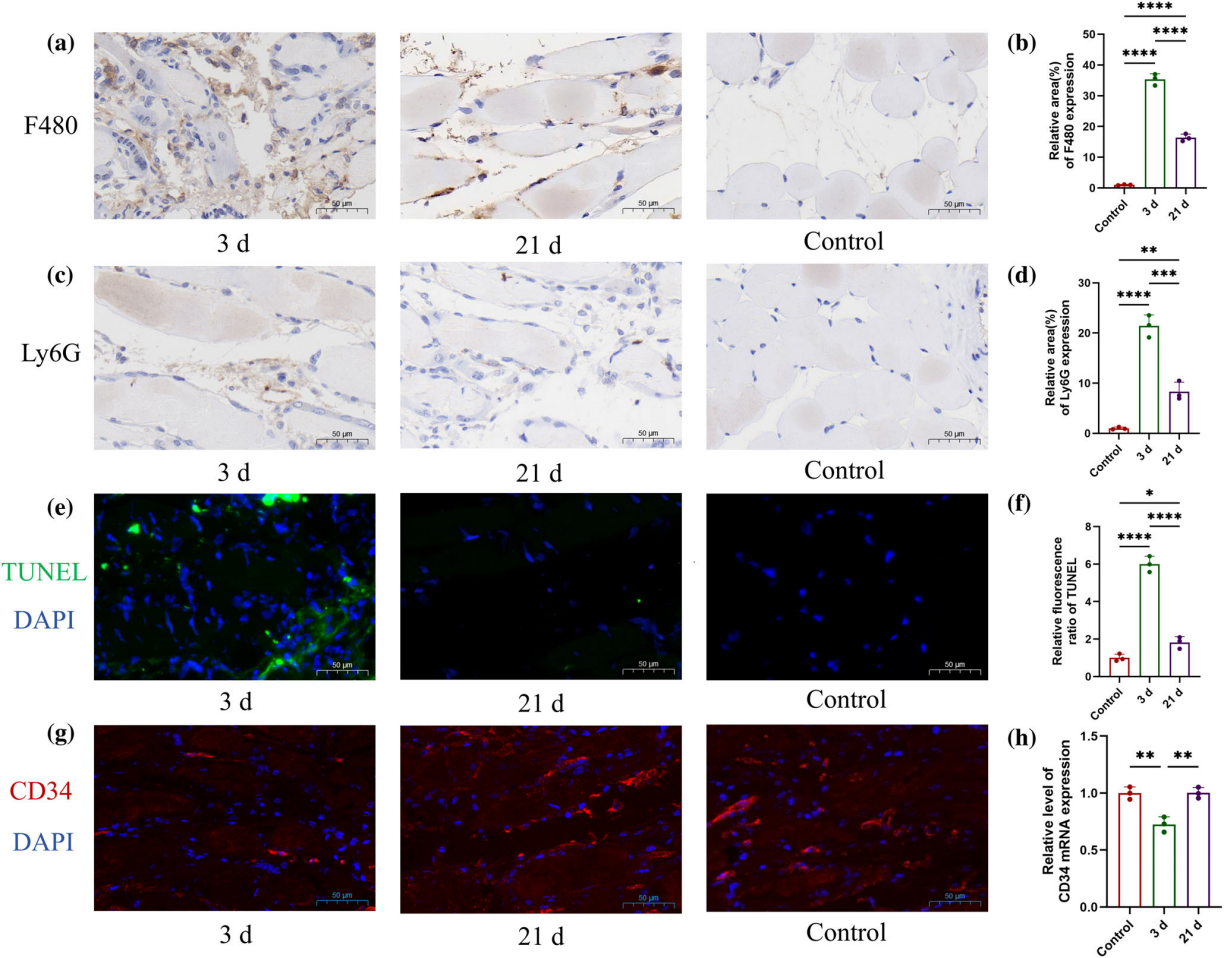
Immunofluorescence and immunohistochemical staining of muscles from the affected limb (3 and 21 days after surgery) and the control group, and quantitative analysis (male c57) (*n* = 5). (a,b) F480. (c,d) Ly6G. (e,f) TUNEL. (g,h) CD34. *p < 0.05, **p < 0.01, ***p < 0.001, ****p < 0.0001.

Immunohistochemical staining of F480 and Ly6G in the muscles of the affected limbs of ALLI mice and the control group, revealed that the expression levels of F480 (Figure 5a,b) and Ly6G (Figure 5c,d) were significantly greater than those of the control group at 3 days after surgery, and decreased at 21 days after surgery but were still greater than those of the control group. These results reflect the trend of postoperative muscle inflammation in the affected limb. TUNEL staining of the muscles of the affected limb and control groups revealed that the relative level of TUNEL increased significantly at 3 days after surgery and almost returned to the control level at 21 days after surgery (Figure 5e,f). This finding indicated that the level of apoptosis in the muscle of the affected limb was high at 3 days after surgery and then gradually decreased. On the other hand, the CD34 fluorescence signal of the muscle of the affected limb decreased significantly 3 days after surgery, and gradually increased to the control level at 21 days after surgery (Figure 5g,h). The decrease in CD34 levels might be due to ischemic and oxidative stress. Increased CD34 levels are due to increased neovascularization.

The results of immunohistochemistry and immunofluorescence staining revealed that the condition of the muscle of the affected limb was very poor at 3 days after the operation, and inflammation and apoptosis were high and gradually recovered at 21 days. This finding was consistent with the muscle stiffness results.

### 2.5 OCTA characterization of the lower limb microcirculation

**FIGURE 6:**
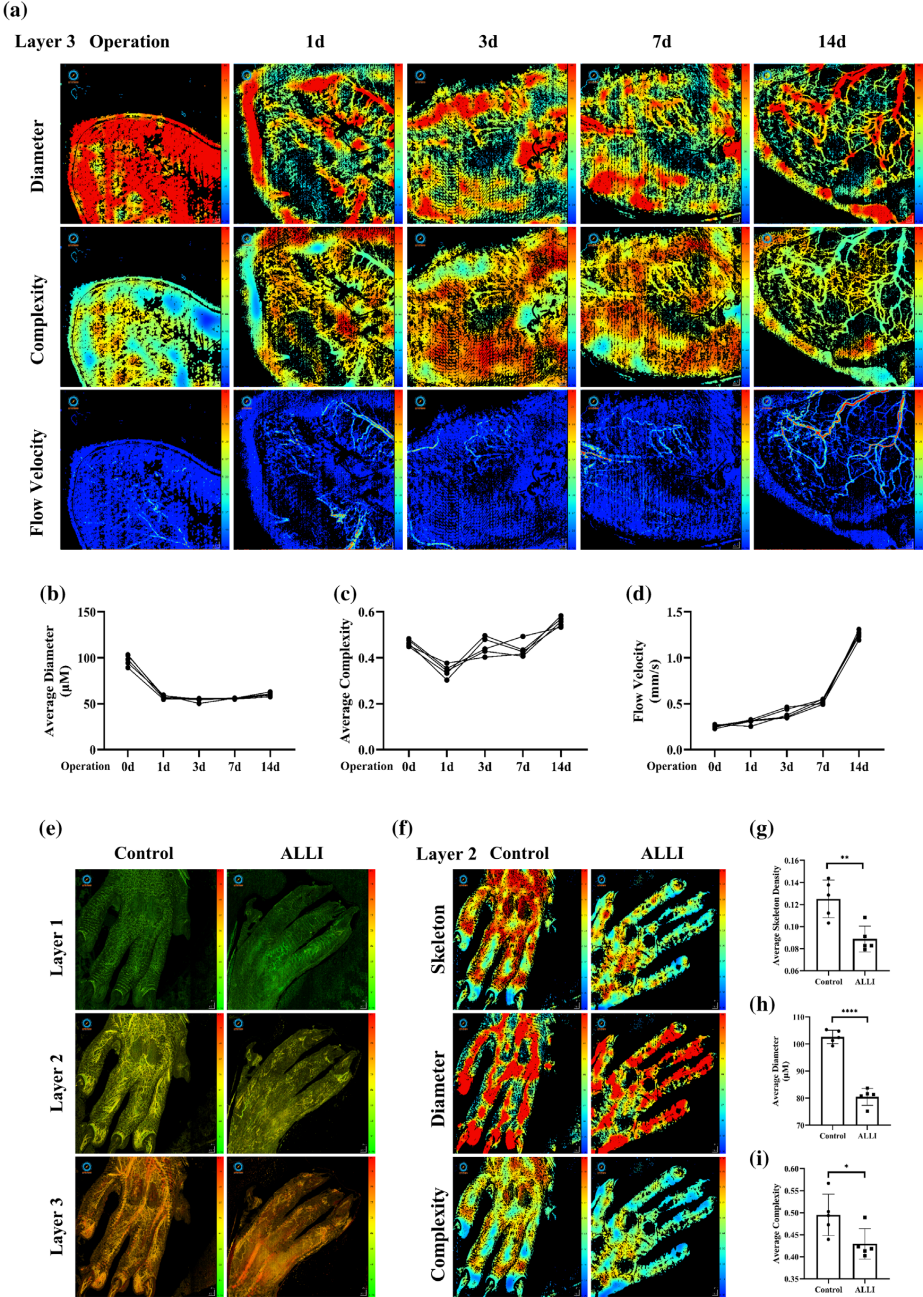
OCTA imaging for the diagnosis and continuous monitoring of ALLI (male C57) (*n* = 5). (a) OCTA imaging of the affected limbs of ALLI mice. Layer 3 (40–100) was selected for testing, and the detection parameters were: Vessel diameter, vessel complexity, and blood flow velocity. The continuity detection time was as follows: Operation (0d), 1, 3, 7, and 14 days after operation. (b–d) Quantitative analysis of vessel diameter, vessel complexity, and blood flow velocity in ALLI mice (*n* = 5). (e,f) OCTA imaging was performed on the dorsum of the feet of control and ALLI 3d mice. Layers (20–40) were selected for detection, and the detection indices used were the vascular skeleton, vessel diameter, and vessel complexity. (g–i) Quantitative analysis of the vascular skeleton, vessel diameter, and vessel complexity in ALLI mice (*n* = 5). *p < 0.05, **p < 0.01, ****p < 0.0001.

OCTA is a revolutionary method for visualizing blood vessels in all layers of the retina as well as the choroid. The microcirculation of the medial gastrocnemius muscle and dorsum of the foot in the affected limb of ALLI mice was examined via the OCTA technique. The backs of normal mice were first imaged by OCTA, which was divided into three layers with depths of layer 1 (0–20), layer 2 (20–40), and layer 3 (40–100) (Figure S20a). The skeleton map, vascular diameter map, and complexity map of layer 3 are shown in Figure S20b. Next, the medial calf of ALLI model mice was imaged, and OCTA images of the three layers are shown in Figure S21. At 3 days postsurgery, the number of microvessels in all layers was significantly reduced and had basically returned to normal at 7 days postsurgery. Next, OCTA images of layer 3 of the medial calf of ALLI mice were selected for quantitative analysis (Figure 6a–d). As a result of FA ligation, there is a massive deficit in the microcirculatory blood supply in the immediate postoperative period, the mean diameter of the vessels decreases dramatically, and the mean diameter of the microcirculation begins to rise as time progresses. The average complexity of microcirculation also shows a fluctuating upward trend. In addition, layers 1 and 2 do not extract valid blood flow velocities because of the inability of ultrasound machines to recognize blood flow velocities in overly thin vessels. In contrast, the flow velocity maps of layer 3 showed that at 7 days postoperatively, perfusion of the affected limb increased, with the appearance of many collateral circulations in which flow velocity could be detected (Figure 6a,d). In addition, owing to difficulties in dorsal fixation and image acquisition, only OCTA images of the dorsum of the foot in normal and ALLI 3d mice are shown (Figure 6e–i). Quantitative analysis revealed that the average density of blood vessels, the average diameter of blood vessels, and the vascular complexity of the dorsum of the foot were significantly lower reduced in ALLI mice than in normal mice.

CD34 immunofluorescence staining was performed on the skin of the affected limb (Figure S22a). At 3 days after surgery, the CD34 levels were significantly lower than those in the control group. After 21 days of operation, the CD34 level clearly recovered and was almost equal to the control level (Figure S22b). This reflected changes in the skin microcirculation of the affected limb: severe impairment at 3 days after surgery and gradual recovery at 21 days. This trend was the same as the change in microvessel density observed by OCTA, confirming the accuracy of the OCTA results.

### 2.6 Comparison of NIR‐II imaging and RFLSI imaging results in different mouse strains

**FIGURE 7:**
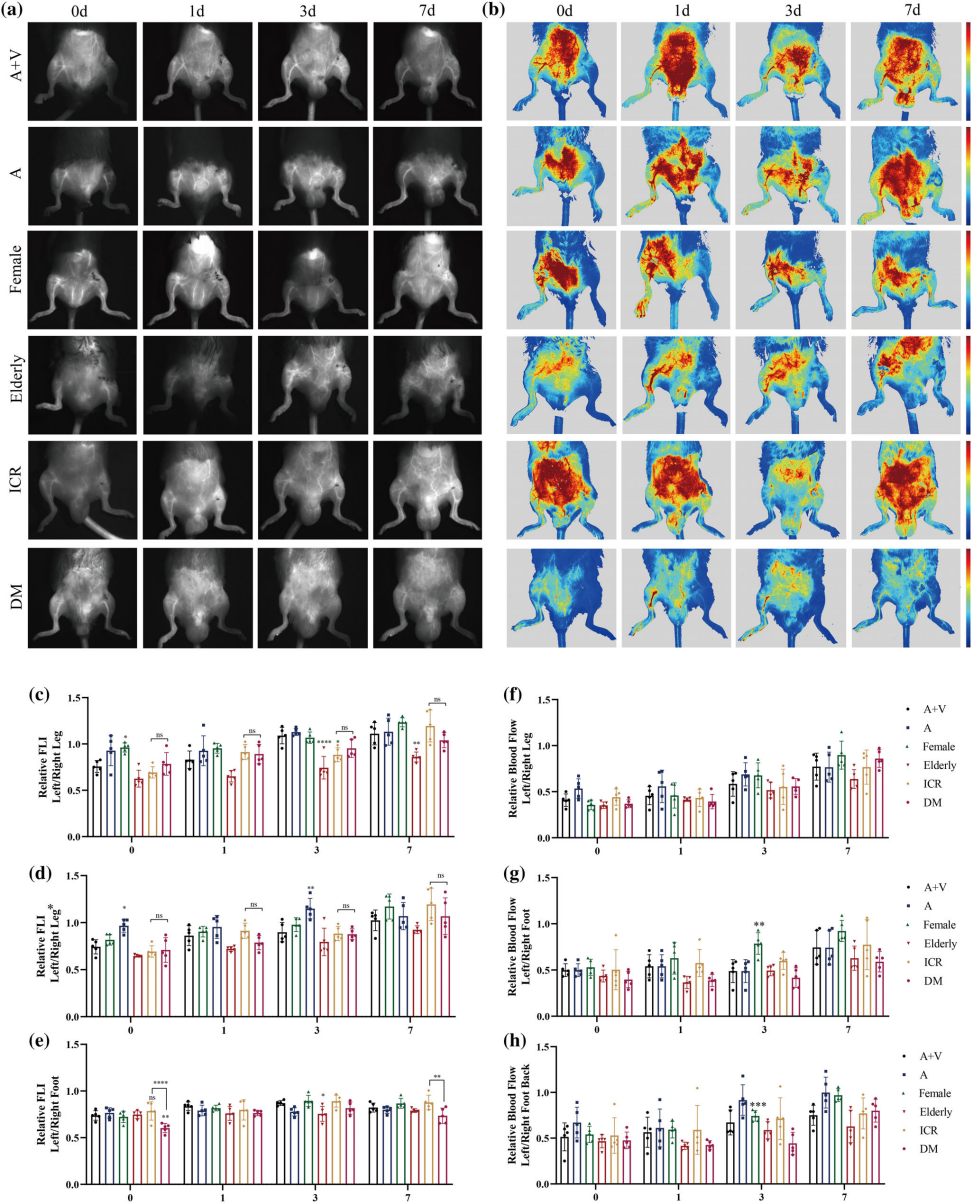
Comparison of NIR‐II imaging and RFLSI imaging results in different mouse strains (male C57‐FA ligation, male C57‐FAV ligation, female C57, T2D, ICR, and elderly mice) at the following time points: 0, 1, 3, and 7 days (*n* = 5). Representative (a) NIR‐II images and (b) RFLSI images at the above strains. The relative FLI of the fluorescence intensity of the left leg to that of the right leg, fluorescence intensity of the left femoral artery to the right femoral artery, and the fluorescence intensity of the left dorsal foot to that of the right dorsal foot in (c–e) NIR‐II images and (f–h) RFLSI images. *p < 0.05, **p < 0.01, ***p < 0.001, ****p < 0.0001.

To test the application value of NIR‐II imaging and RFLSI imaging in different strains of mice, male C57‐FA ligation, male C57‐FAV ligation, female C57, T2D, ICR, and elderly mice were selected for validation. Figure 7a,b shows representative NIR‐II images and RFLSI images at the above strains. Surprisingly, NIR imaging revealed reduced lower limb perfusion in the ALLI model in aged and T2D mice (Figure 7c–e). These results indicated that advanced age and T2D could reduce lower limb perfusion levels in mice. Quantitative analysis of the RFLSI results revealed similar results (Figure 7f–h). These findings indicated that these two imaging methods were suitable for different mouse strains to some extent, and the corresponding perfusion characteristics could be determined.

### 2.7 Application of RFLSI imaging, NIR‐II imaging, and DUS imaging in humans

**FIGURE 8:**
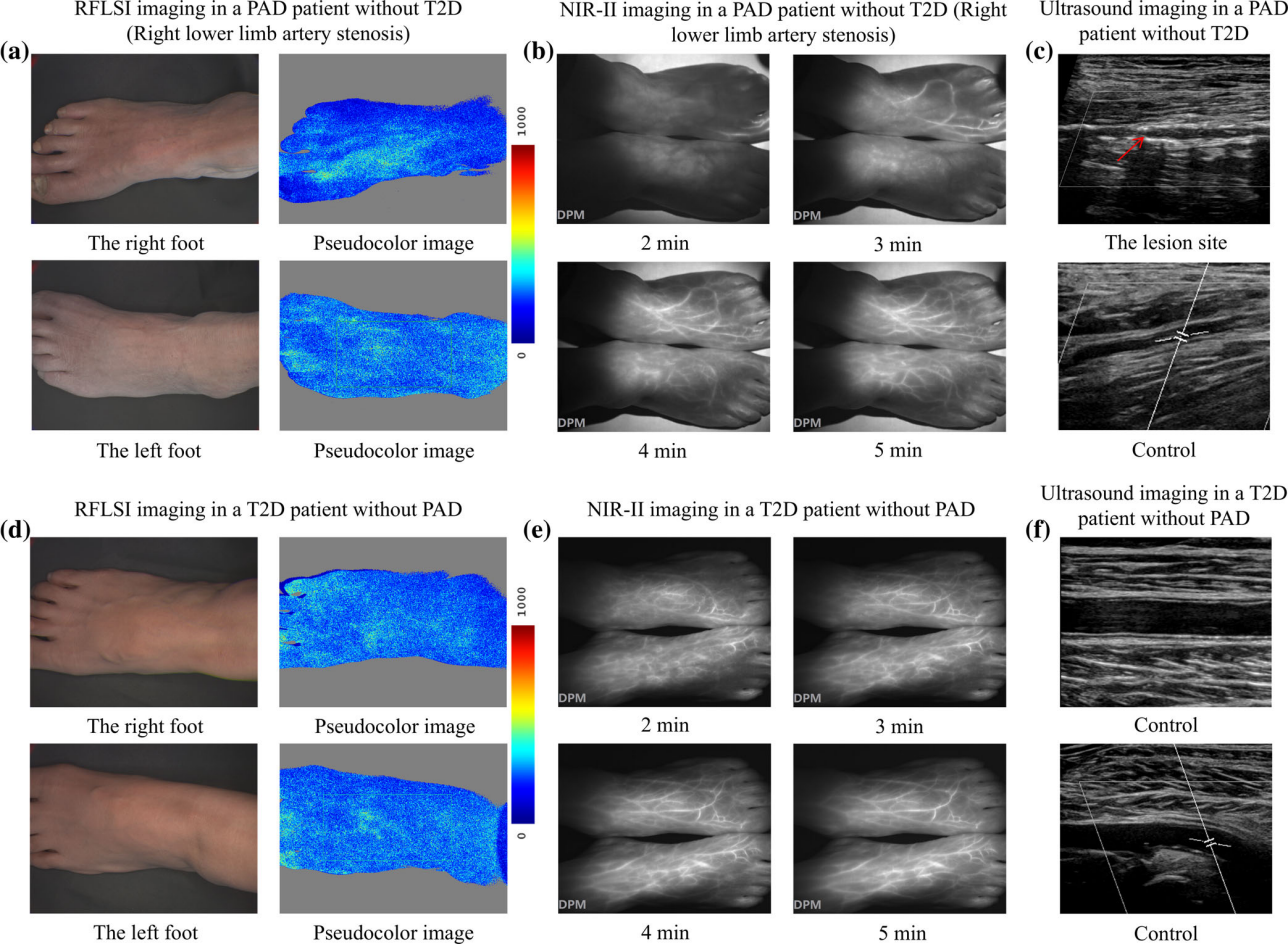
Application of RFLSI imaging, NIR‐II imaging and DUS imaging in humans. (a) RFLSI imaging, (b) NIR‐II imaging, and (c) DUS imaging in a PAD patient without T2D. (d) RFLSI imaging, (e) NIR‐II imaging, and (f) DUS imaging in a PAD patient without T2D.

In this study, RFLSI imaging, NIR‐II imaging, and DUS imaging were applied in a PAD patient without T2D and a T2D patient without PAD to examine their potential application in humans. Figure 8a–c shows the application of these three imaging methods in a PAD patient without T2D, with right lower extremity artery occlusion. RFLSI imaging revealed that perfusion was significantly lower in the right foot than in the left foot (Figure 8a). At 2 and 3 min, fluorescence appeared on the left foot, but not on the right foot. At 4 and 5 min, the blood vessel density of the left foot was greater than that of the right foot. This finding confirmed that perfusion of the left foot was stronger than that of the right foot. DUS revealed plaque in the right lower limb artery and patency in the left lower limb artery. Figure 8d–f shows the application of these three imaging methods in a T2D patient without PAD. RFLSI imaging revealed almost identical perfusion in both feet. NIR‐II imaging revealed that fluorescence appeared in both feet at 2 min, and the blood vessel density and intensity of both feet were almost the same. DUS demonstrated arterial patency in both lower limbs. No adverse reactions occurred during any of the imaging processes. These findings demonstrated the potential and safety of these imaging techniques for human applications.

## 3 DISCUSSION

In the present study, we established a stable and effective ALLI model using combined FA ligation, and for the first time, this model was comprehensively evaluated in terms of the vascular occlusion plane, blood flow, tissue ischemic injury, de novo collateral circulation formation, and histopathology by a combination of RFLSI, NIR‐II, US, and OCTA.

According to the 2016 AHA/ACC Guidelines on the Management of Patients With Lower Extremity Peripheral Artery Disease,^24^ accurate perfusion assessment is essential for the diagnosis and treatment of ALLI, as well as facilitating timely hemodialysis. Currently, ankle brachial index (ABI) measurement of the lower dorsal foot or posterior tibial ankle or toe brachial index (TBI) measurement is an important method to characterize perfusion of the lower limbs, and it is widely used for the diagnosis and prognosis of PAD.^25^ However, its sensitivity is decreased in patients with small‐vessel disease due to hypertension, diabetes, or chronic kidney disease.^26^, ^27^ In addition, techniques such as CTA and MRA, which target the assessment of vascular anatomy in the lesion area have limitations of high cost and susceptibility to complications, making them unsuitable for mass screening of high‐risk populations.^28^ Therefore, finer and more comprehensive perfusion data are needed to assess the severity of ALLI and to predict treatment response.

LSI relies on the unique light absorption properties of hemoglobin to visualize the microvascular system, providing real‐time, dynamic, in vivo, noncontact monitoring of changes in lower limb blood flow with high temporal and spatial resolution, with no need for contact with body tissues and the use of tracers,^29^ which is uniquely suited for applications in both clinical and research scenarios. For example, LSI was used by Wido Heeman et al. in laparoscopic surgery to identify ischemic intestinal tubes with microcirculatory injuries to reduce the incidence of anastomotic fistulas^30^; and LSI was used to establish a mouse model of acute hypoxia and transient middle cerebral artery occlusion to visualize cerebral blood flow in deep subcortical regions.^31^ In the present study, the RFLSI was used to quantify the decline in and recovery of lower limb perfusion in the affected limb of mice with ALLI. After acute ischemia, impaired blood flow was spontaneously attenuated and essentially normalized at Day 7. Bryan Wermelink et al.^32^ reported improved clinical outcome parameters when LSI perfusion values were elevated after hemodialysis in 30 patients with chronic limb‐threatening ischemia (CLTI). Indeed, part of the perfusion we measured with LSI comes from the microcirculation from the skin, which is affected by lower limb ischemia.^33^ Using LSI, Johanna Berggren et al. assessed the hemodynamic reconstruction of skin grafts and flaps after surgery.^34^ In addition, LSI can be used to quantify acute cutaneous vascular permeability responses and characterize inflammatory skin diseases associated with increased blood flow.^35^ This finding is consistent with our animal model results.

In recent years, NIR‐II imaging has become an emerging technology for comprehensive diagnosis and image‐guided therapy, and with the advantages of high imaging resolution, low background, low autofluorescence, and deep‐tissue imaging capability, a variety of probes have been developed to identify microscopic breast cancers,^36^ hepatocellular carcinomas,^37^, ^38^ high‐grade gliomas,^39^ and other lesions. In this study, NIR‐II angiography using ICG accurately identified the plane of blood flow truncation, and revealed significant obstruction of both arterial perfusion and venous return in the lower extremity on the side of ALLI. Surprisingly, we observed neoplastic collateral vessels in the affected limb of ALLI in a mouse model. Braun et al. previously used indocyanine green angiography (ICGA) to assess perfusion in patients with PAD in whom the ABI and TBI were not measurable due to vascular calcification by quantifying objective data such as starting intensity, increasing in intensity from baseline to peak intensity, and ending intensity.^40^ In addition, Igari et al.^41^ also reported good correlations between data obtained by the ABI and ICGA data. Yuan Ma et al.^42^ developed a new NIR‐II probe (PSMA@IR1048 NPs) responsive to pathologic hypochlorhydria in a mouse model of ischemia–reperfusion injury of the lower limb. The quantitative assessment of wound tissue viability can accurately predict postoperative skin necrosis in patients with chronic limb‐threatening ischemia who undergo ICG NIR imaging within 5 days of amputation.^43^ These cases illustrate the value of the use of ICG in the quantitative analysis of lower extremity perfusion. Within a safe dose, ICG can be injected multiple times to help surgeons monitor lesion progression or recovery before and after ALLI. However, in practice the fluorescence intensity of NIR‐II is more susceptible to a variety of factors such as ambient light intensity, the distance between the camera and the tissue, the dose of ICG, and the obese body size of the animal, and a small number of allergic individuals need to be test‐sensitized first, which may be a cause for concern.^44^

Ultrasonography has long been a commonly used test for the assessment of acute and chronic lower limb ischemia and is noninvasive, nonradioactive, inexpensive, and reproducible. Strain elastography is used to explore the mechanical and elastic properties of muscle tissue and can provide insight into the level of perfusion in the examined area. Lower muscle elasticity values are a sign of decreased perfusion.^45^ This study not only directly demonstrated the morphologic changes in the lower limb vessels during ALLI injury but also indirectly reflected the changes in perfusion during ALLI lesions by assessing muscle stiffness in the ischemic region, with skeletal muscle stiffness being the highest at 3 days postinjury and basically returning to normal at 7 days. In this study, we also assessed muscle stiffness in the ischemic region, which was the highest at 3 days postinjury. Mariya Maslarska et al. explored lower limb muscle dynamics in patients with veno‐arterial extracorporeal membrane pulmonary oxygenation (VA‐ECMO) using muscle ultrasound transverse wave electrograms (SWEs) for the early identification of patients at high risk of lower limb ischemia and reported that muscle elasticity was decreased and stiffness was increased in high‐risk patients,^46^ which is consistent with our findings. However, US is limited by the level of experience of the examiner, and its accuracy varies when multiple stenoses are present in the same region.

OCTA is a new noninvasive imaging technique commonly used in retinal clinics for rapid, layer‐by‐layer resolution of anatomical structures and vascular systems to quantitatively assess retinal thickness, morphology, and blood flow.^47^ OCTA was applied to monitor vascular changes in the superficial microcirculation at different levels of the ischemic hindlimb, with a decrease in the mean vessel diameter at 1 day due to a reduction in direct perfusion of small vessels. However, the exact mechanism of the blood flow changes was not clear because of the establishment of a de novo collateral circulation network, complexity, and blood flow velocities that were elevated at 14 days postinjury. Previously, OCT has been applied to femoropopliteal endovascular interventions, endovascular imaging of atherosclerosis to insualize microclamping, microthrombosis, and microintegration,^48^ as well as macrophage infiltration, lipid accumulation, in‐stent calcification, and neointimal rupture.^49^ In contrast to OCT, OCTA can be repeated in a single imaging procedure and obtain comprehensive, wide‐field information about the neoplastic collateral circulation, which demonstrates the value of potential clinical applications.^22^ However, OCTA requires an elongated run time and may produce more types of artifacts.^50^

By simulating both arterial and venous obstructions, our model reveals early hemodynamic changes and tissue responses in ischemia. These findings suggest that multimodal imaging, such as laser speckle and near‐infrared spectroscopy, could be valuable for detecting ischemia and evaluating treatment efficacy in human patients. Moreover, real‐time visualization of blood flow and tissue perfusion dynamics could aid clinicians in making more informed decisions regarding interventions and treatment strategies, ultimately improving the management and prognosis of ALLI patients.

A limitation of the present study is that we demonstrated only the dynamic evolution of trunk vascular circulation obstruction and the establishment of de novo collateral circulation during ALLI but did not explore in depth the mode of collateral artery formation^51^ or the mechanism of formation. In addition, multimodal imaging equipment and specific operations need to be further standardized so that reproducible and accurate parameters related to lower limb perfusion can be determined, which is necessary for their clinical dissemination. In conclusion, many of the technologies in the imaging systems described above are in their infancy. Optical imaging is limited by the depth of light penetration to visualize only superficial blood flow, whereas acoustic imaging is not intuitive. However, combining these methods allows comprehensive visualization and quantification of vascular changes in the lower extremities and enhances our ability to detect the severity of ALLI and predict treatment response, which will greatly assist in clinical decision making.

## 4 CONCLUSIONS

In conclusion, this study is the first to combine four optical and acoustic imaging devices, RFLSI, NIR‐II, US, and OCTA, to rapidly and accurately diagnose ALLI in mice and to provide a noninvasive and quantitative strategy to monitor pathological processes in the cutaneous microcirculation, muscle, and collateral circulation. This multimodal integrated imaging approach shows great promise in the diagnosis and monitoring of ALLI, as well as in the assessment of treatment options and prognosis.

## 5 MATERIALS AND METHODS

### 5.1 Animals and ALLI model

The ALLI model was constructed in male C57, female C57, T2D, ICR, and elderly mice (40 w). The methods used for mouse selection and ALLI model construction were described in the Supporting Information. The steps of ALLI model construction are shown in Figure S23. *N* = 5 was used for each group in the experiments according to previous publications.^52^, ^53^

### 5.2 RFLSI imaging and analysis

The abdomen and both lower limbs of each mouse were imaged using a real‐time wide‐field laser scatter flow imaging system (SIM Opto‐Technology Co., Ltd., Wuhan, China). When the microvessels were illuminated by the laser, the reflected laser intensity varied due to the continuous flow of red blood cells, resulting in a fuzzy speckle image on the image sensor. By contrast analysis of the speckle image, the perfusion image could be obtained. The perfusion image was printed to the gray range of 0–255 to form a gray image, and the gray image was endowed with pseudocolor to obtain a pseudocolor image. Prior to RFLSI imaging, the mice were achieved by a small animal anesthesia machine (TAIJI‐IE, RWD Life Science Co., China) using isoflurane. Hair was removed from the abdominal and bilateral lower limbs, and the mice were placed on the RFLSI imaging platform in the supine position. The abdomen and both lower limbs of the model and control mice were imaged using the RFLSI acquisition system, with the mice placed 40 cm below the detector. The focus was adjusted to obtain clear blood flow images, and all photographs were taken at the same zoom (40×), exposure time (5 ms), and pseudocolor threshold settings. The RFSI images were analyzed using RFLSI analysis software. Both lower limbs of the selected mice were quantified for perfusion. During RFLSI image acquisition and analysis, all examiners were unaware of the grouping of the imaged mice. RFLSI imaging was performed on each mouse before and 1, 2, 3, 5, 7, 10, 14, and 21 days after the model was created.

### 5.3 NIR‐II imaging and analysis

The experimental mice were depilated on the abdomen and both lower limbs, and gas anesthesia was performed using isoflurane. Afterward, 200 μg of ICG (0.1 mg/mL) was injected into the tail vein of the imaged mice, and NIR‐II fluorescence imaging was performed using an InGaAs SWIR camera (Xenics Cheetah‐640CL TE3) coupled with an NIR‐II transmissive lens (Spacecom VF50M SWIR), with a filter wheel fixed on the front of the lens (1100 nm, USA) used to collect NIR‐II fluorescence. The excitation laser wavelength ranged from 808 nm, the power was set to 10,000 mW, and the exposure time was 0.5 to 2 s. The bilateral anterior thigh and bilateral dorsal foot were used as regions of interest (ROIs). The contrast within the ROIs in the NIR‐II images was quantified by calculating the fluorescence intensity. The ROIs were analyzed with the built‐in analysis software External Data Processing to generate a time–intensity curve. NIR‐II imaging was done on each mouse before, on the day of, and 1, 2, 3, 5, 7, 10, 14, and 21 days after the model was created.

### 5.4 Ultrasound imaging and analysis

Two‐dimensional ultrasound imaging, microcirculatory perfusion imaging, and strain elastography (STE) were performed using a V700VE ultrasound system and an SL3116 (12–25 MHz) probe (Esaote Medical Co., Ltd., China). STE was evaluated by manual compression. There was a phase difference between the echo signal before and after compression. By solving the phase difference and converting it into the displacement difference, the deformation caused by compression could be calculated. All the radio‐frequency signals of all the particles were converted into strain values and presented with color coding. Tissues with small elastic coefficients and large displacement changes after compression were shown in red. Tissues with high elastic coefficient and small displacement change after compression were shown in blue. Tissues with moderate elasticity were shown in green. Mouse hair was removed using depilatory cream prior to ultrasound scanning, and then a warm ultrasound gel was applied to the inner left thigh. During ultrasound imaging, the mice were anesthetized with isoflurane, and B‐mode ultrasound images, C‐mode ultrasound images, and STE images were acquired by placing the mice in a supine position on the scanning platform and monitoring their physiological conditions including cardiac and respiratory cycles. The PSV, end diastolic velocity (EDV), and RI were extracted from the images (VINNO ULTIMUS 9LAB). Ultrasound imaging was done on each mouse 1, 3, and 7 days after the model was created.

### 5.5 OCTA imaging and analysis

Prior to imaging, the mice were anesthetized with isoflurane using a small animal anesthesia machine. Next, the mice were wrapped with a sponge block to prevent movement and maintain body temperature. The mice were placed on the imaging platform with continuous gas anesthesia. Saline was added so that the lower limb to be imaged fit closely to the lens. Microvascular imaging of the mouse lower limbs was performed using a vascular microcirculation in vivo monitoring system (Micro‐VCC, Optoprobe, UK), which uses a swept source laser with a central wavelength of 1060 nm and an A‐scan rate of 200 kHz. Scanning was performed using the OPTO‐III scanning software, and the pixel data block size corresponding to an OCTA scan was 600 × 600 × 1280 × 20, indicating that each A‐scan contained 1280 pixels, each B‐scan contained 600 A‐scans, and each C‐scan contained 600 B‐scans. When we perform OCTA scanning, we need to repeat the scanning 20 times on each B‐scan, which is extracted by the OCTA algorithm to obtain the 3D microvascular signal. For the blood flow velocity scan, 200 repetitions were performed for each scan point, and then the corresponding points were extracted according to different time intervals. Finally, the machine learning model was used to construct the flow velocity distribution map. The acquired data were subjected to vascular signal extraction by the analysis software Pyoct 8.0. Points with blood flow signals generate signal perturbations, and we identify them as blood vessels. Signal extraction is then completed and image generation is carried out, in which different colors are used to represent different depths. The image is quantitatively analyzed by software to obtain data related to the density of the vascular skeleton and the complexity of the vessels. OCTA imaging was performed on each mouse before and 1, 2, 3, 5, 7, 10, 14, and 21 days after the model was created.

### 5.6 Immunofluorescence staining and TUNEL staining

Immunofluorescence (IF) staining TUNEL was performed on paraffin sections of mouse skin and muscle tissue. Localization of the target proteins was performed as follows: dewaxing, antigen repair, and serum blocking. The primary antibody was then added and incubated overnight at 4°C in a wet box, followed by fluorescent labeling. The nuclei were then stained with DAPI. Terminal deoxynucleotidyl transferase‐mediated nick end labeling (TUNEL) (Servicebio, Wuhan, China) was performed on muscle tissue. Anti‐CD34 (Servicebio, Wuhan, China) was used to perform IF staining of skin and muscle tissue.

### 5.7 Histology and immunohistochemistry analysis

Paraffin‐embedded tissue sections (5‐μm‐thick) were stained with hematoxylin and eosin (H&E). For immunohistochemistry (IHC), anti‐F480 (Servicebio, Wuhan, China) and anti‐Ly6G (Servicebio, Wuhan, China) were used to incubate the slices. Pathologic and IHC images were obtained using a light microscope (Pannoramic MIDI, 3DHISTECH Ltd., Hungary).

### 5.8 Statistical analysis

GraphPad Prism 7 software was used for statistical analysis. The experimental data were expressed as the mean ± standard deviation (SD). The Kolmogorov–Smirnov test was used to verify whether the data followed a normal distribution. For statistical analysis, a *t*‐test was performed for normally distributed data and Mann–Whitney *U*‐test was executed for nonnormally distributed data. One‐way ANOVA and two‐way ANOVA were used for comparisons among multiple groups when staining was quantified and imaging results were compared between groups. *p* < 0.05 was considered statistically significant.

## PROTOCOL REGISTRATION STATEMENT

A protocol (including the research question, key design features, and analysis plan) was prepared before the study, and this protocol was registered by the Ethics Committee of the Second Hospital of Shanxi Medical University (No. DW2023053, [2024]YX214).

## AUTHOR CONTRIBUTIONS

**Heng Wang:** Conceptualization; data curation; methodology; investigation; formal analysis; project administration; visualization; writing – original draft; writing – review and editing. **Keyi Fan:** Methodology; data curation; investigation; validation; formal analysis; visualization; writing – original draft. **Yijie Ning:** Data curation; investigation; methodology; validation; visualization; formal analysis; writing – original draft. **Chuanlong Lu:** Methodology; data curation; investigation; writing – original draft. **Yuhang Zhang:** Methodology; data curation; investigation. **Qian Wang:** Methodology; data curation; visualization. **Hongjiu Zhang:** Data curation; visualization; methodology. **Yaling Li:** Data curation; methodology; visualization. **Zeyu Zhang:** Methodology; writing – review and editing. **Xiaohua Jia:** Methodology; writing – review and editing. **Sheng Yan:** Methodology; writing – review and editing. **Ruijing Zhang:** Resources; project administration; supervision; writing – review and editing. **Honglin Dong:** Funding acquisition; project administration; supervision; resources; writing – review and editing.

## CONFLICT OF INTEREST STATEMENT

The authors have no conflicts of interest to declare.

## PEER REVIEW

The peer review history for this article is available at https://www.webofscience.com/api/gateway/wos/peer-review/10.1002/btm2.70005.

## Supporting information


**Data S1.** Supporting Information.

## Data Availability

Data sharing not applicable to this article as no datasets were generated or analysed during the current study.
