# Supplementary material for: Noninvasive monitoring of vascular alterations in mice with acute lower limb ischemia using multimodal photoacoustic imaging
Source: Bioeng Transl Med. 2025 Feb 17;10(4):e70005. doi: 10.1002/btm2.70005 (PMC12284427; doi:10.1002/btm2.70005)
Supplement: Supplementary file 1 — Data S1. Supporting Information. [file BTM2-10-e70005-s001.pdf]

Supplementary Methods

Animals and acute lower limb ischemia model

Eight-week-old SPF-grade male C57 , female C57, male ICR mice were purchased from Nanjing Junke Bioengineering Co. The experimental unit is a single mouse. Streptozotocin (STZ)-treated ICR mice were used to create a model of type 2 diabetes (T2D). After four weeks of high-fat feeding, STZ diluted with citrate buffer was injected through the tail vein at doses of 60, 80, 100, 80, and 80 mg/Kg for five consecutive days. T2D modeling was considered successful when random blood glucose exceeds 16.1. The experimental unit was a single animal.

The ALLI model was created through the following steps: after anesthetizing the mice in the supine position with isoflurane, the mice were shaved bilaterally, moistened with iodine volts, and an incision at the level of the femoral artery was made along the medial side of the thighs, approximately 5 mm long. In the ALLI group, the femoral artery and femoral vein was dissected and freed, and the proximal and distal ends of the femoral artery and femoral vein were ligated with a 10-0 suture above the artery on the abdominal wall, respectively, and later cut through the middle with surgical scissors. To explore the value of multimodal imaging in different disease states, two modeling protocols were used in male C57 mice: femoral artery and vein (FAV) ligation, and only femoral artery (FA) ligation.

The ALLI model (n=5, the total number of animals used=36) was constructed in male C57 (FAV ligation), male C57 (FA ligation), female C57, T2D, ICR and elderly mice (40w). Criteria used for inclusion of mice: successful modeling and survival for at least 21 days after modeling. Criteria used for exclusion of mice: failure of modeling, death of mice within 21 days after modeling, and persistent wounds on the skin of lower limbs. One C57 male mouse was excluded as it did not survive until 21 days after modeling. The exact value of n in male C57 mice was 6. The exact value of n in other groups was 5. In this study, all experimental mice were raised under the same feeding conditions and living environment, and 2-3 mice were raised in each cage. Only the first author was aware of the group allocation at the different stages of the experiment,

and the imaging operation was mainly done by other authors. All mice were euthanized after the last imaging. All animal experiments were approved by the Ethics Committee of the Second Hospital of Shanxi Medical University (No. DW2023053).

Supplementary Figure Legends

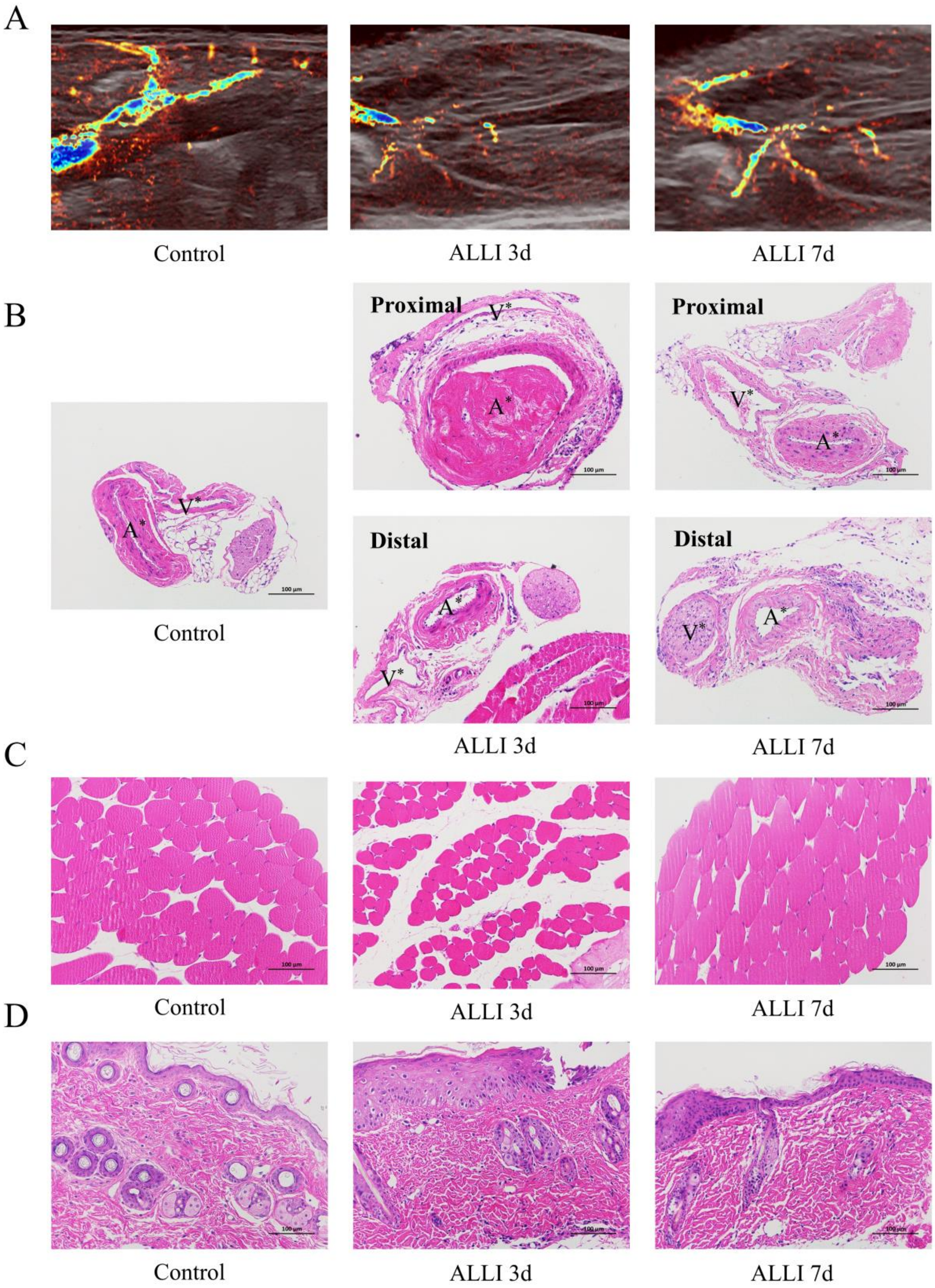

Figure S1: Ultrasound results and pathologic features evaluate model building in ALLI mice after operation 3d, 7d and control group. (A) Ultrasound imaging was performed in ALLI mice. **(B)** H&E staining of

blood vessels in the lower limbs. **(C)** H&E staining of skeletal muscle muscles in the legs of mice. For H&E staining of lower limb skin, the thickness of epidermal layer 3 days after modeling was much higher than that of control group, and there were more inflammatory cells. 7 days after modeling, the thickness of the epidermal layer decreased, but the inflammatory cells was still more than that of the control group.

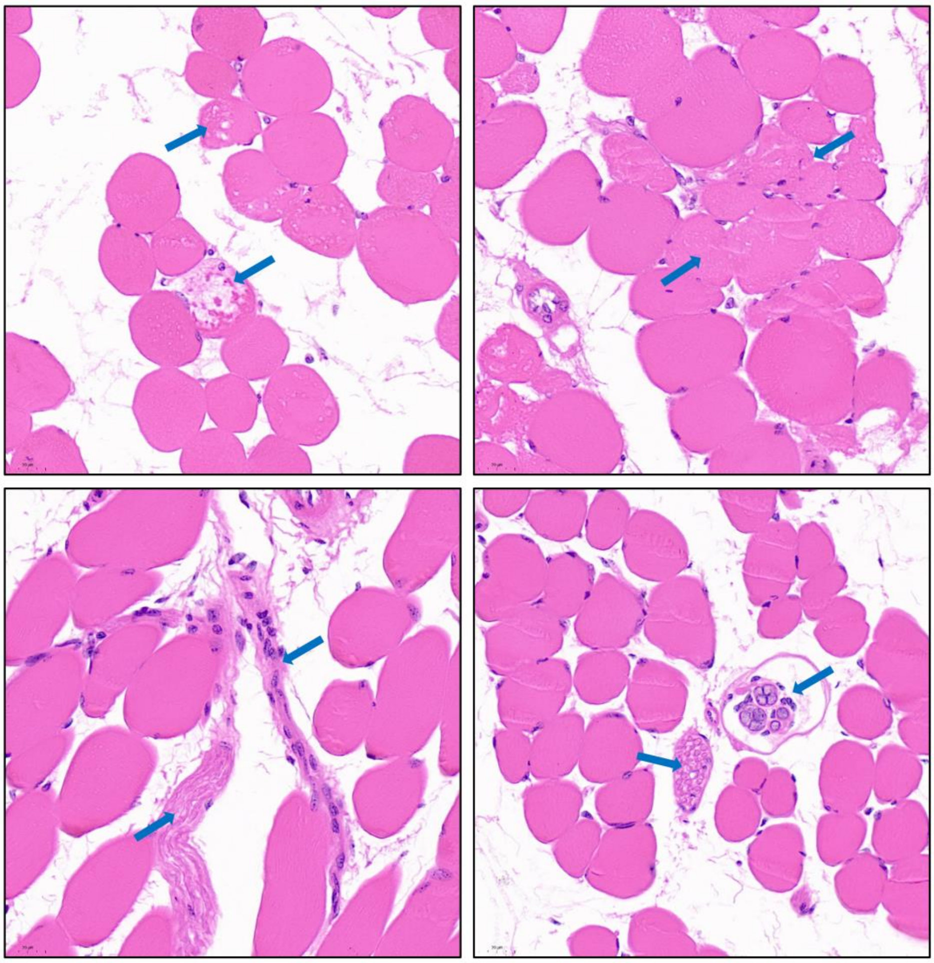

Figure S2: Skeletal muscles of affected limbs of ALLI 3d mice were stained with H&E. Arrows point to: disintegrating skeletal muscle cells, collagen secretion and inflammatory cell infiltration.

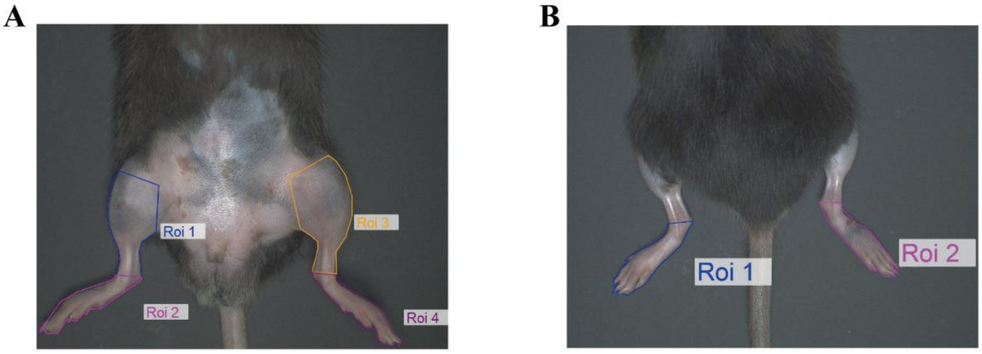

Figure S3: Three areas were selected to quantify RFLSI imaging. (A) Bilateral legs (Roi1 and Roi3 in A) and both feet (Roi2 and Roi4 in A). **(B)** Bilateral dorsal foot (Roi1 and Roi2).

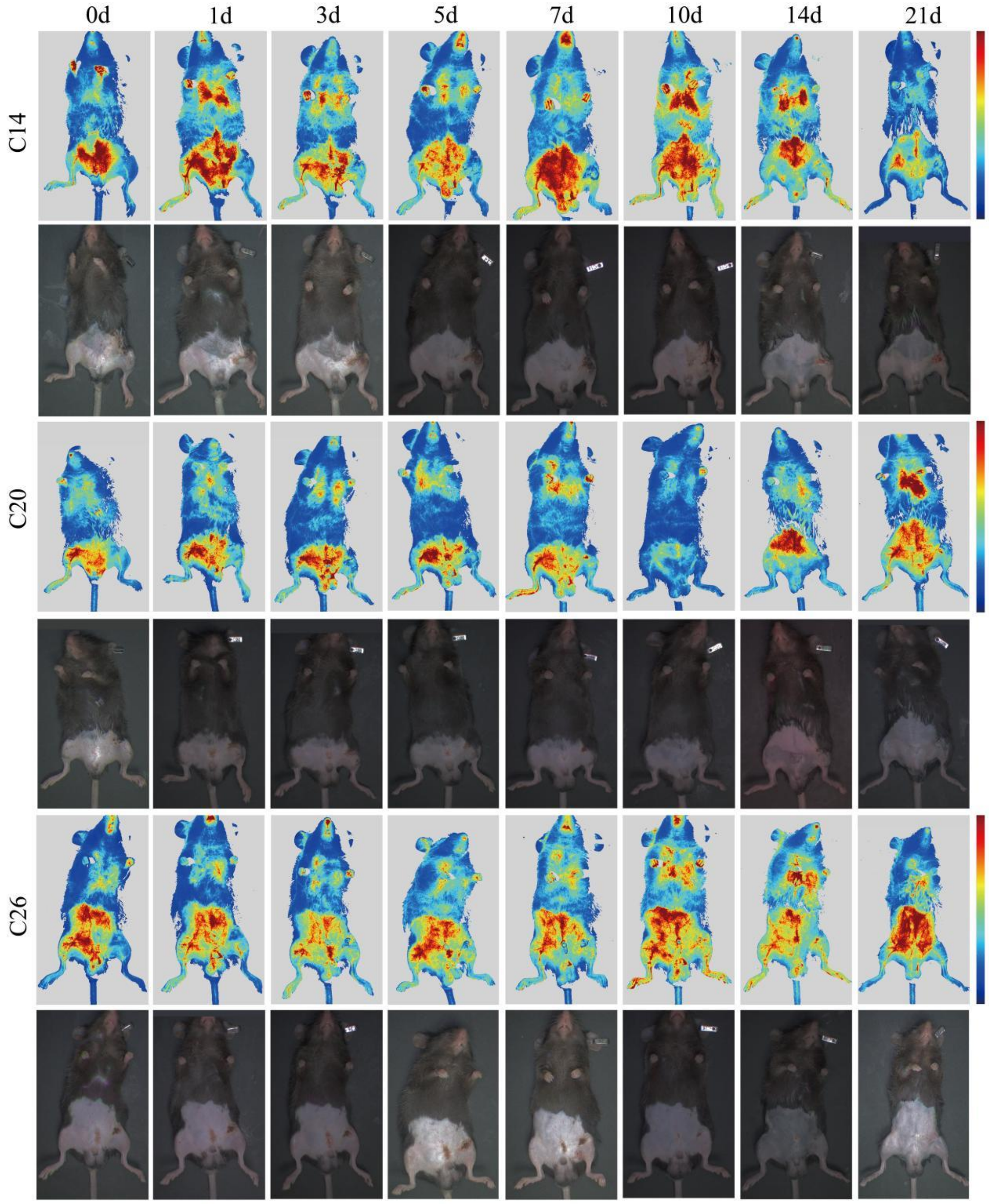

Figure S4: Detailed mice photos and RFLSI images in male C57 minc-femoral artery (FA) ligation (the front).

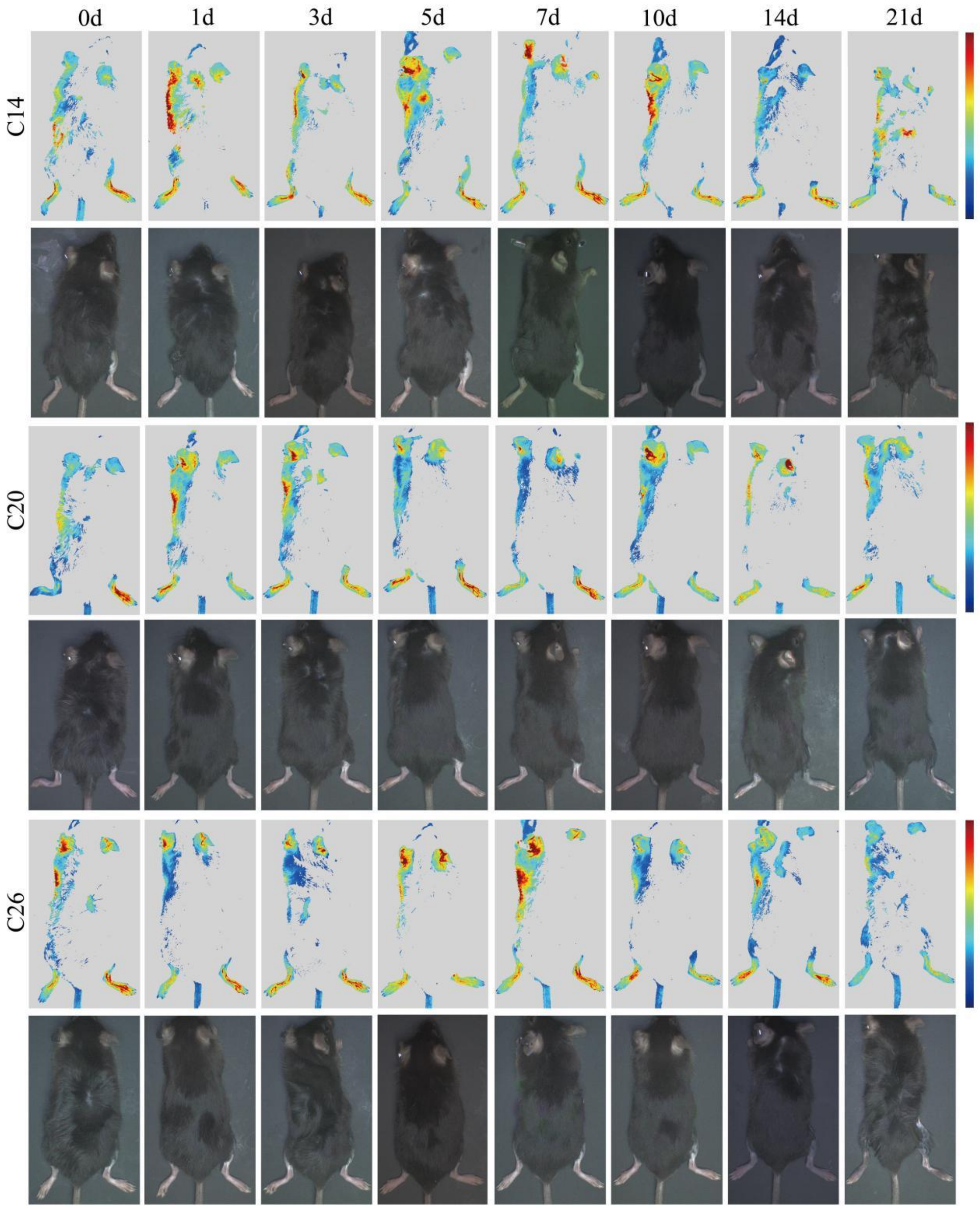

Figure S5: Detailed mice photos and RFLSI images in male C57-femoral artery (FA) ligation (the back).

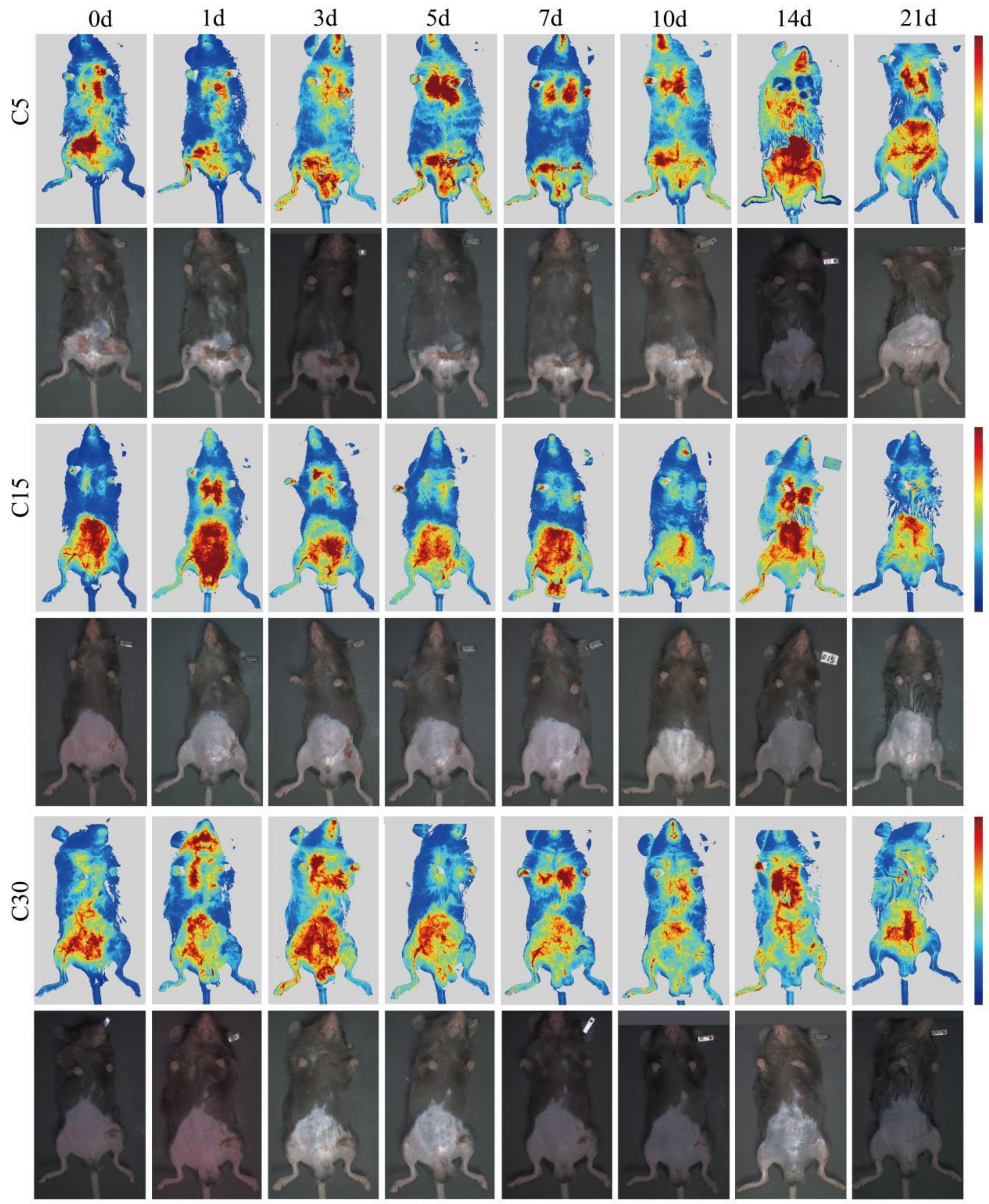

Figure S6: Detailed mice photos and RFLSI images in male C57 mice-femoral artery and vein (FAV) ligation (the front).

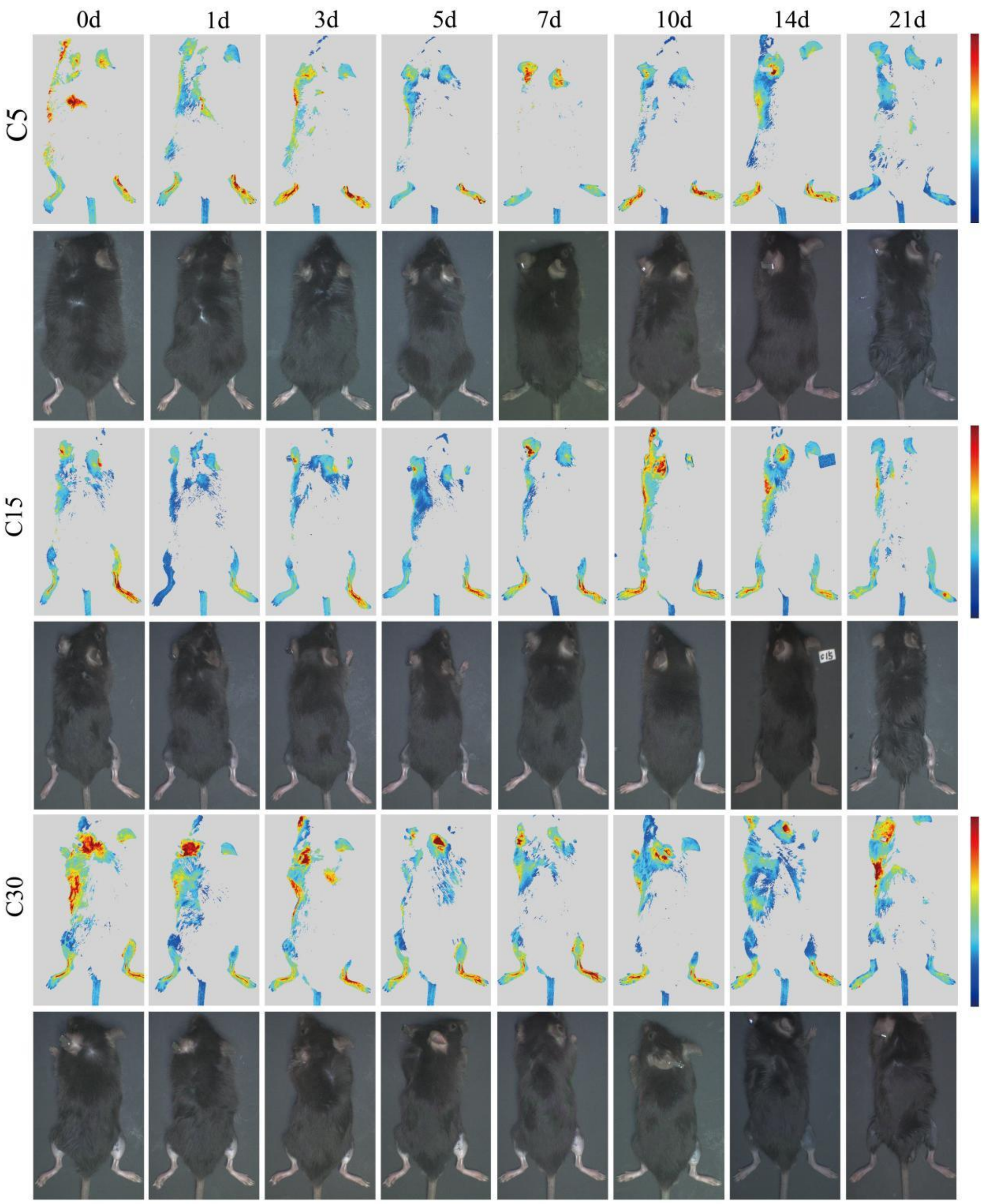

Figure S7: Detailed mice photos and RFLSI images in male C57 mice-femoral artery and vein (FAV) ligation (the back).

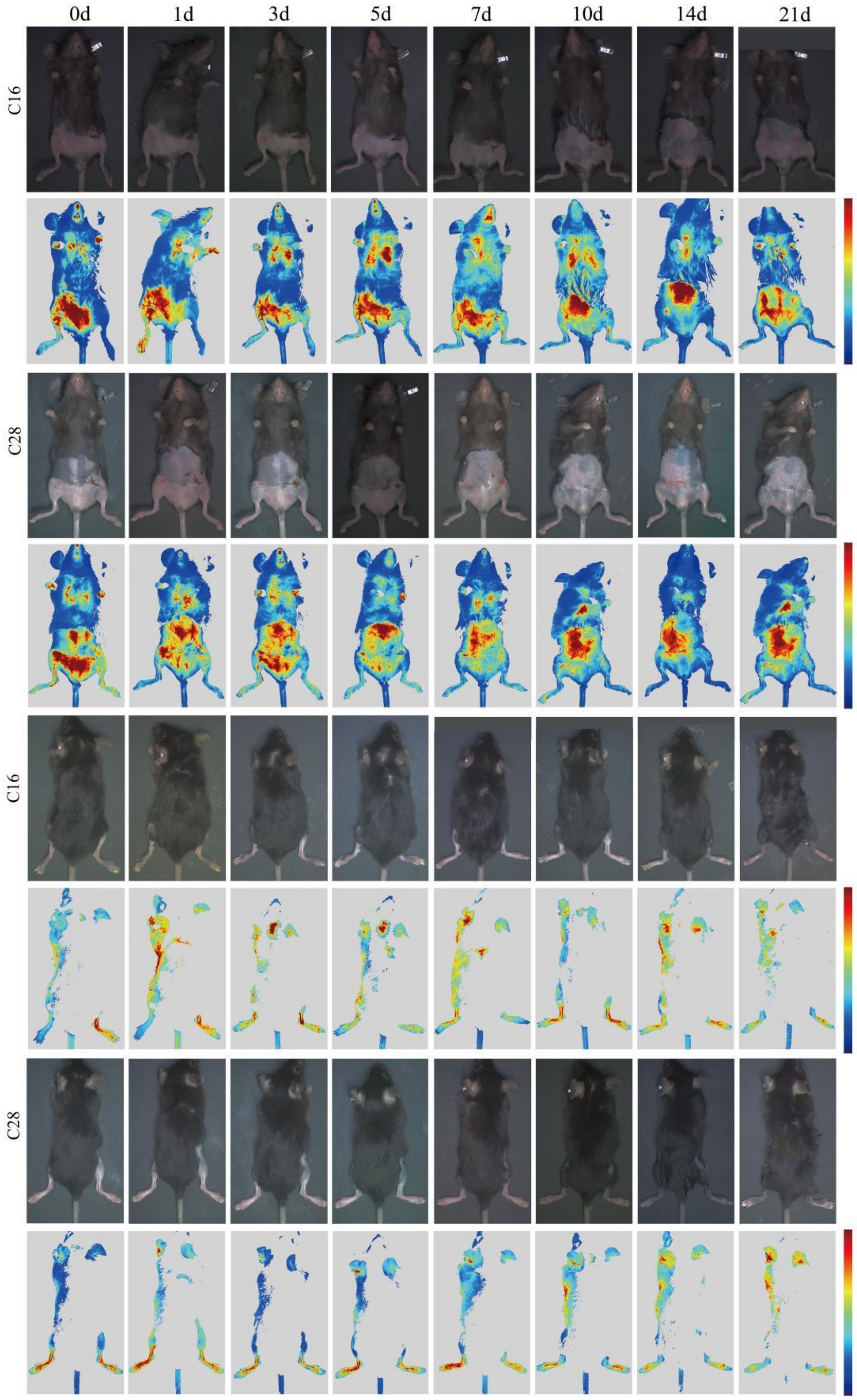

Figure S8: Detailed mice photos and RFLSI images in female C57 mice.

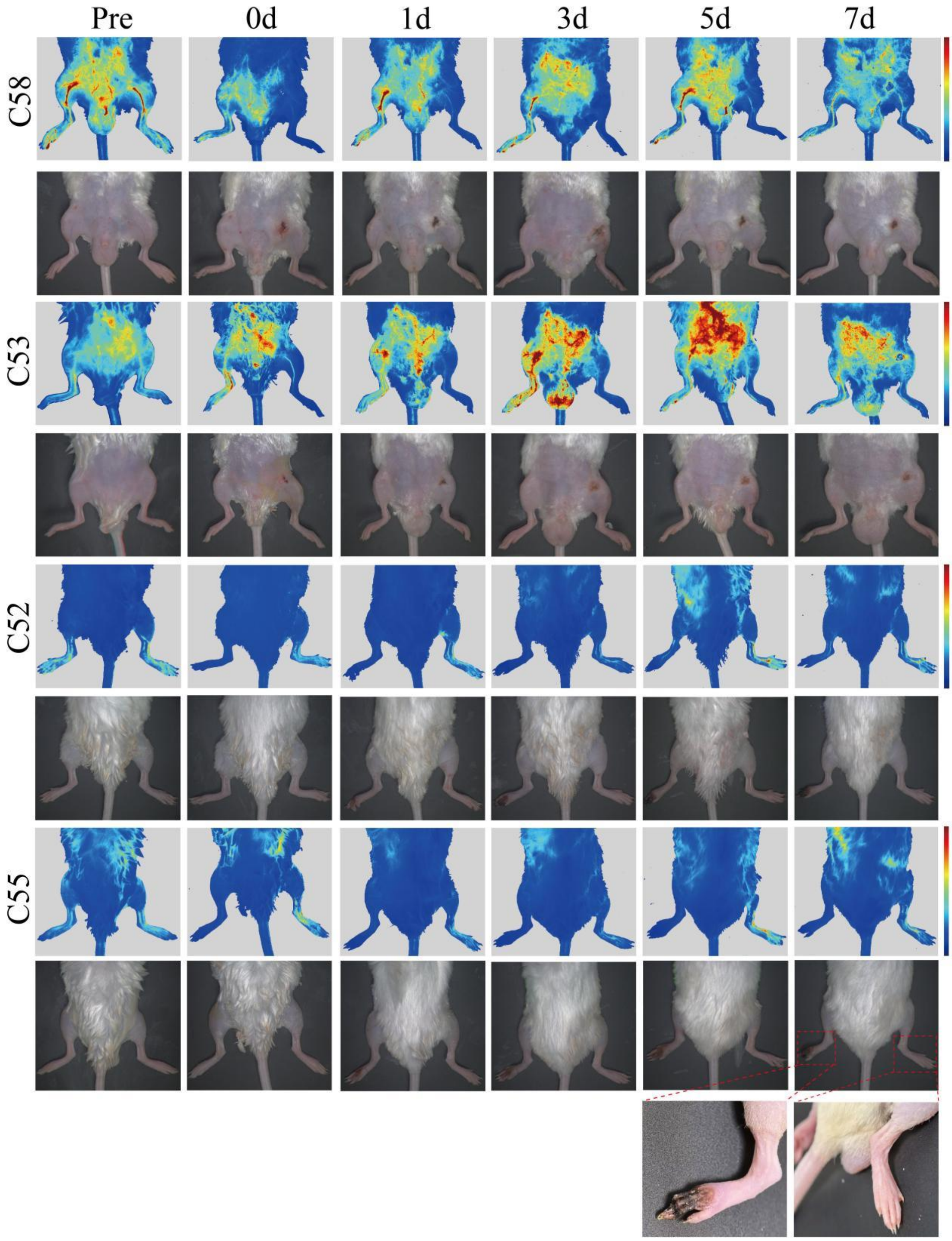

Figure S9: Detailed mice photos and RFLSI images in T2D mice. The last line showed that the mice had ulcers on the left foot and no ulcers on the right foot.

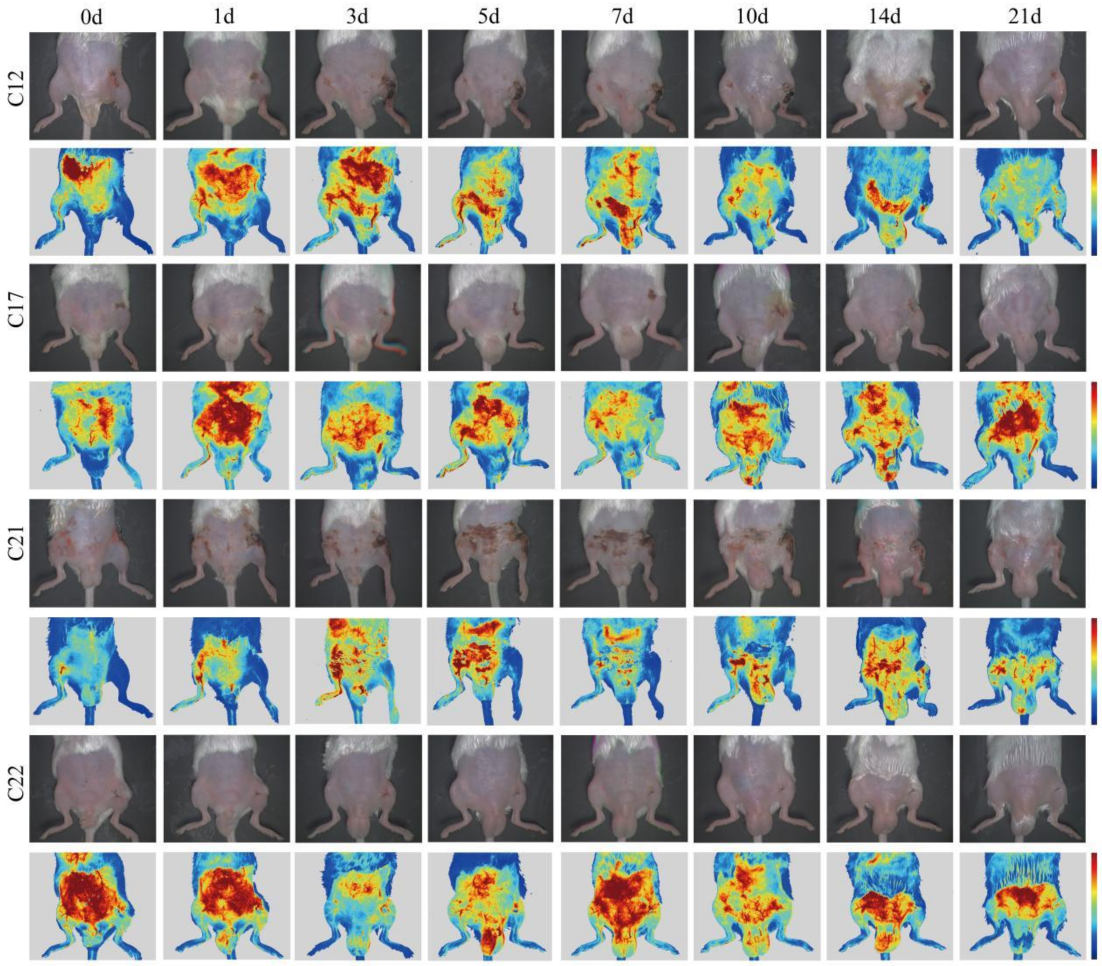

Figure S10: Detailed mice photos and RFLSI images in ICR mice (the front).

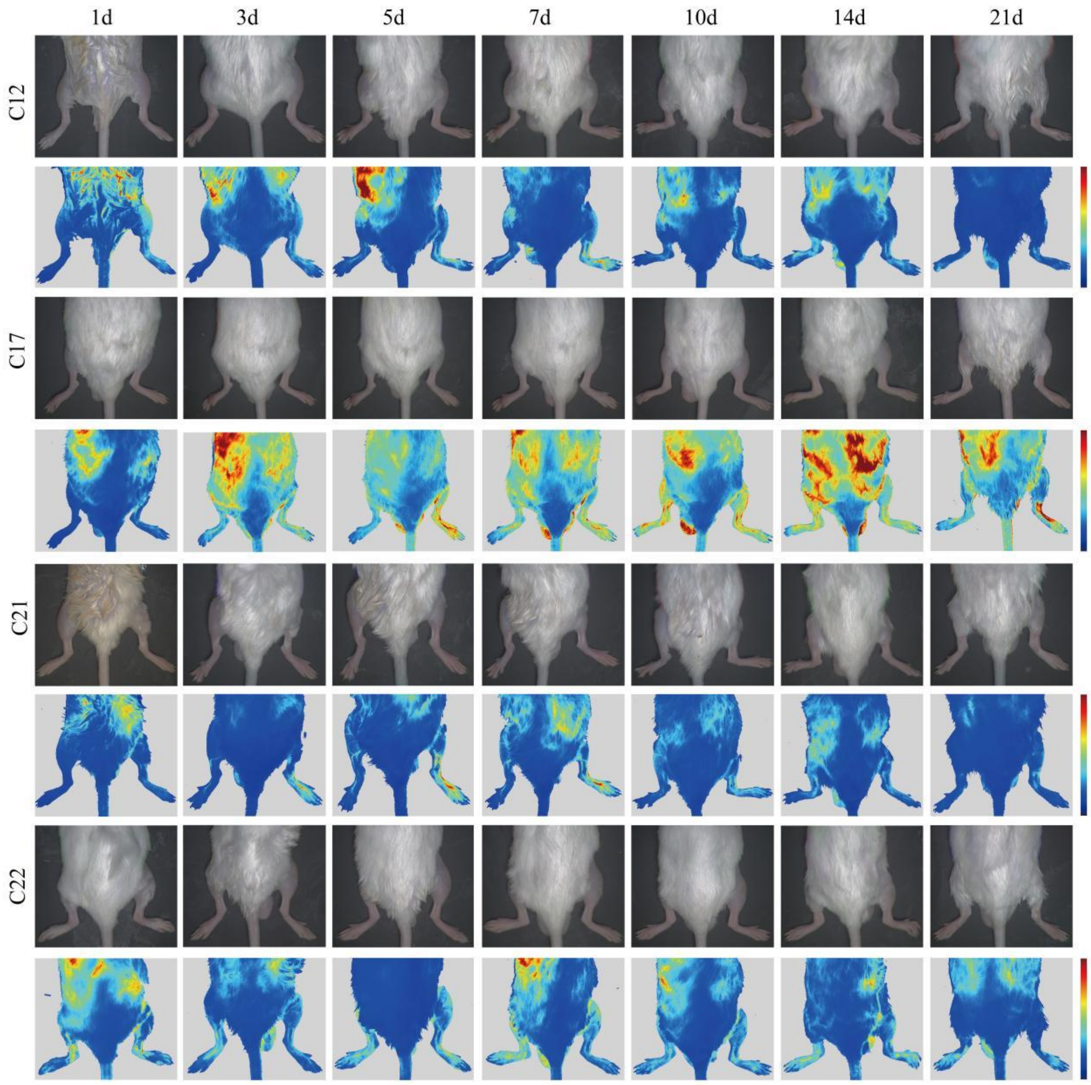

Figure S11: Detailed mice photos and RFLSI images in ICR mice (the back).

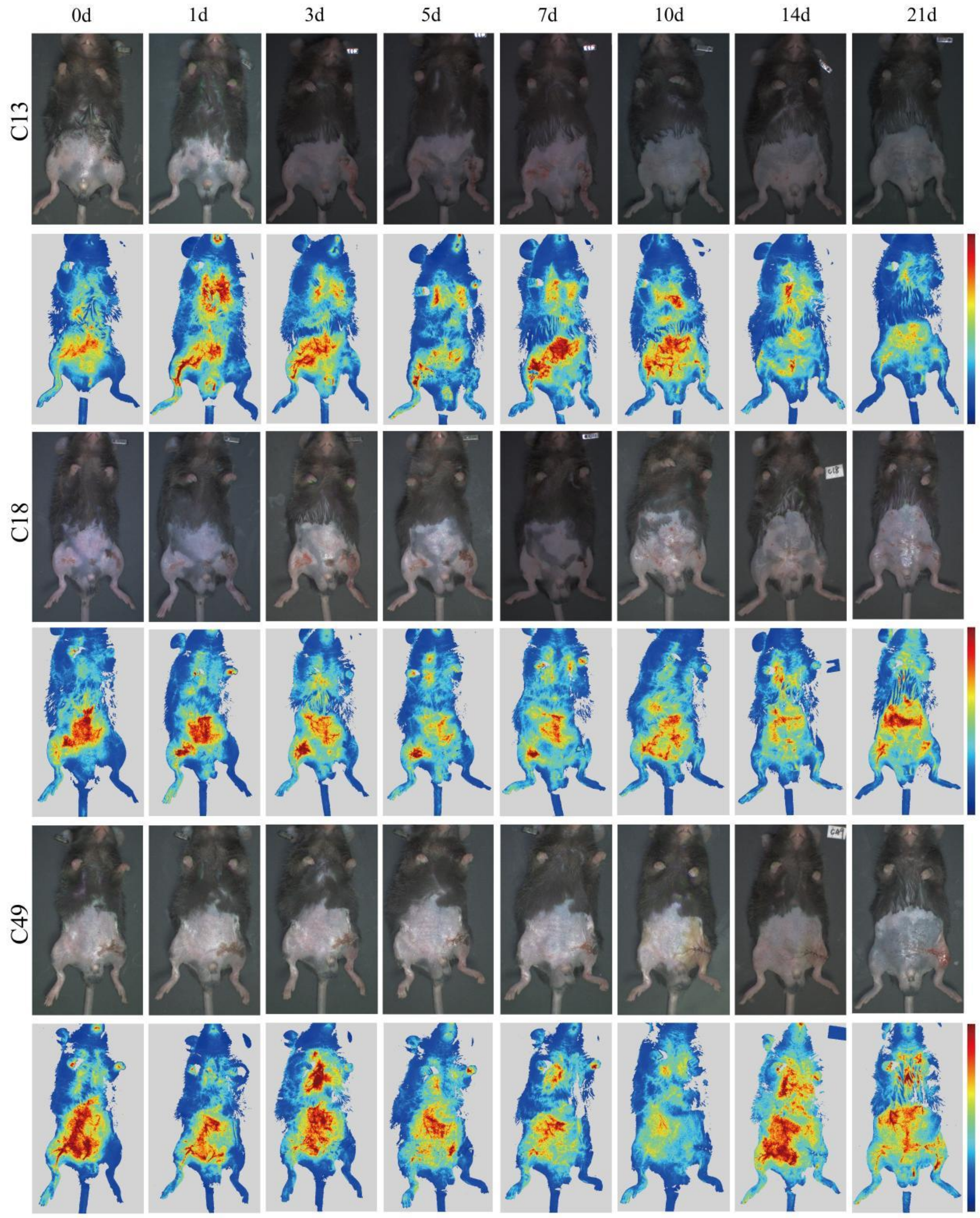

Figure S12: Detailed mice photos and RFLSI images in elderly mice (the front).

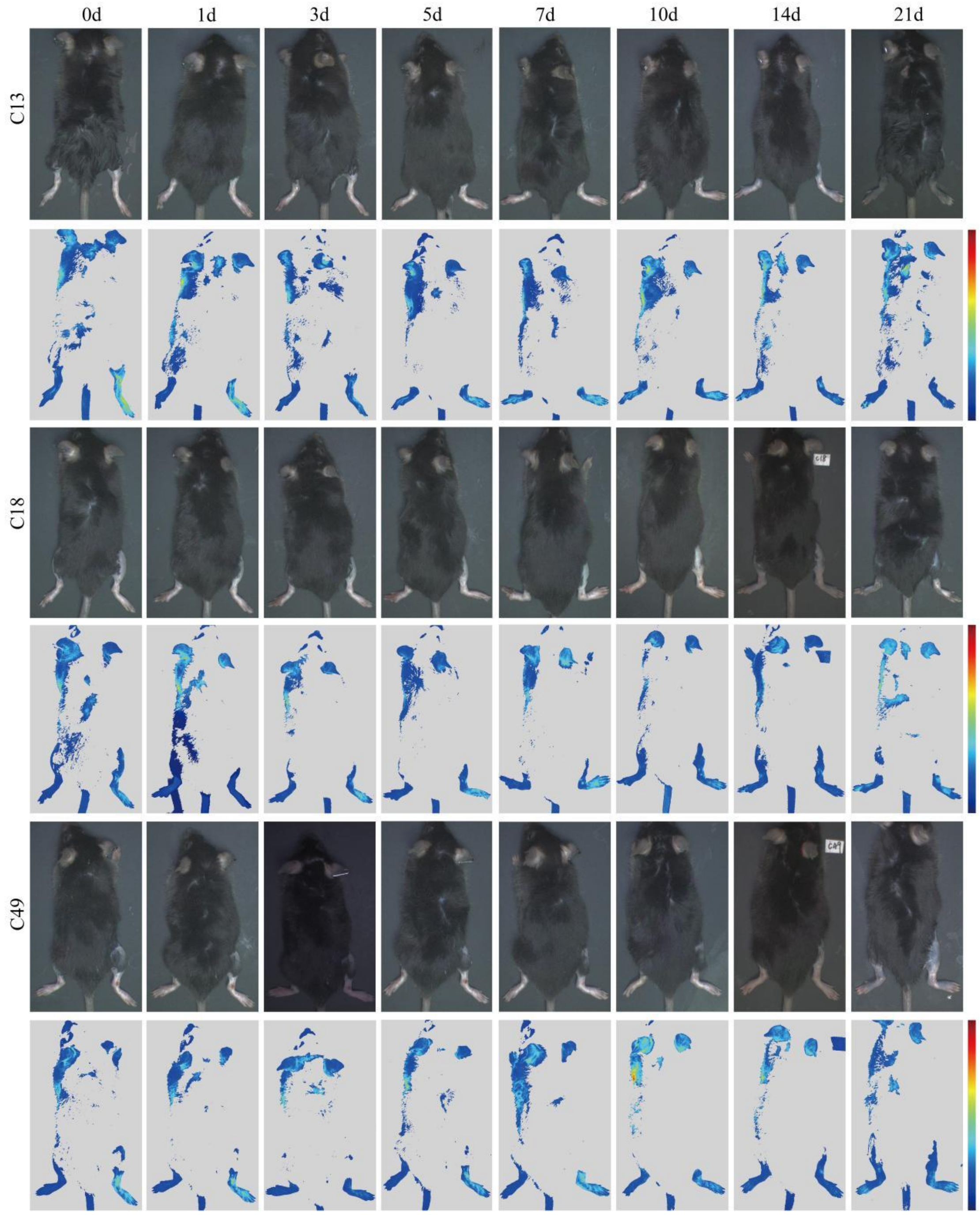

82 **Figure S13: Detailed mice photos and RFLSI images in elderly mice (the back).**

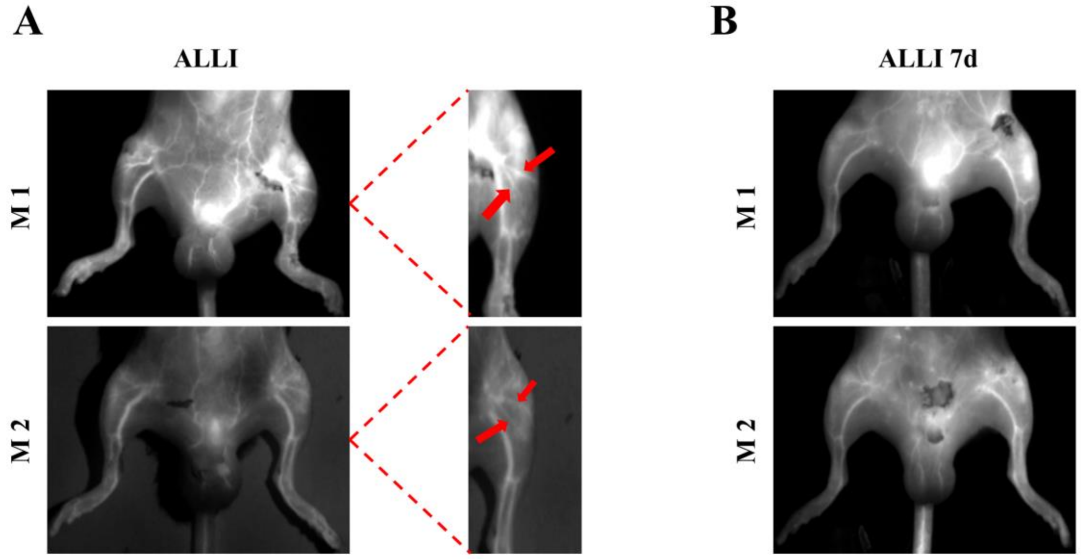

Figure S14: Prognostic monitoring of ALLI mice by NIR-II imaging. **(A)** ICG-based NIR-II imaging identifies collateral circulation in the lower limb of ALLI mice. **(B)** NIR-II imaging was performed on ALLI 7d mice and the intensity of lower limb vascular imaging was similar on both sides.

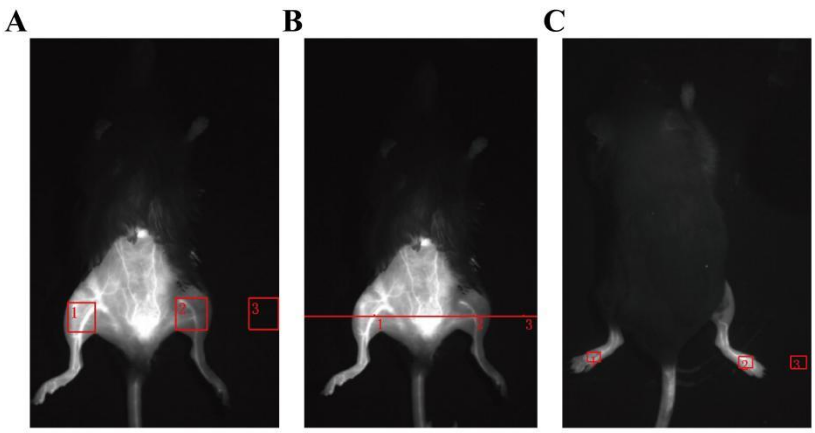

Figure S15: Three areas were selected to quantify NIR intensity. (A) Bilateral legs. **(B)** Bilateral points at femoral artery. **(C)** Bilateral dorsal foot.

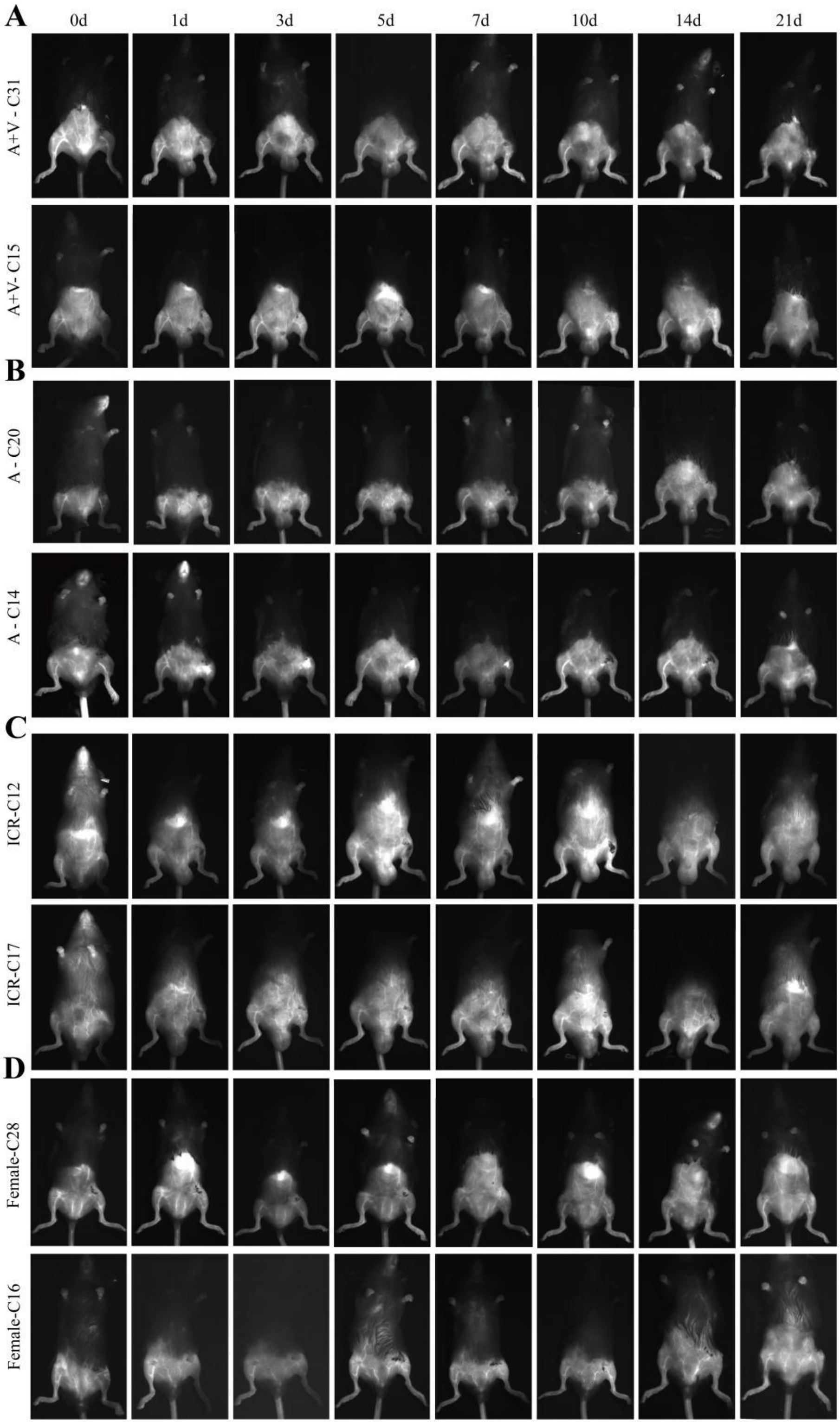

Figure S16: Representative NIR-II images in (A) male C57 mice-femoral artery and vein (FAV) ligation, (B) male C57 mice-femoral artery (FA) ligation, (C) ICR C57, and (D) female mice.

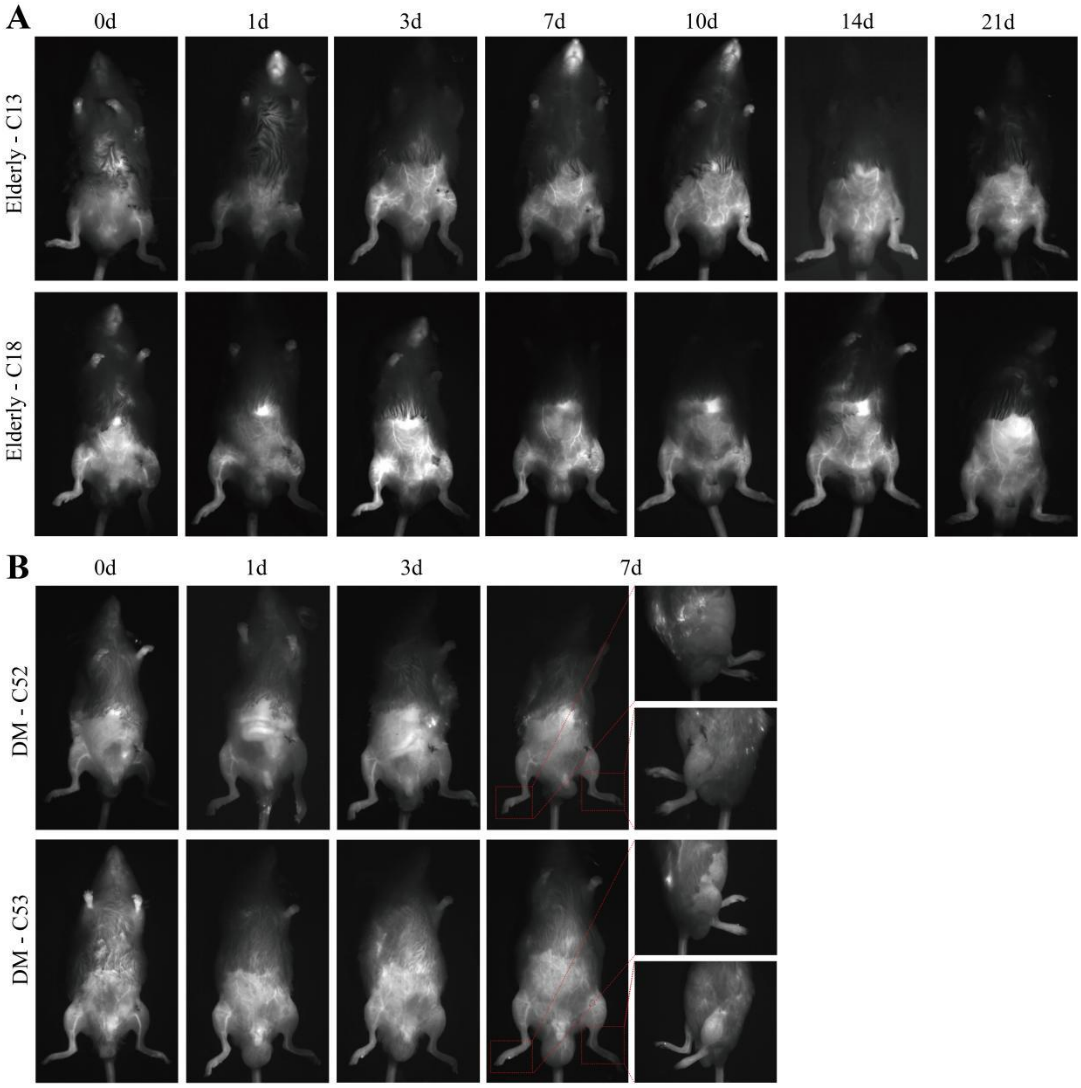

99

100 **Figure S17: Representative NIR-II images in (A) Elderly mice and (B)**

101 **DM mice.**

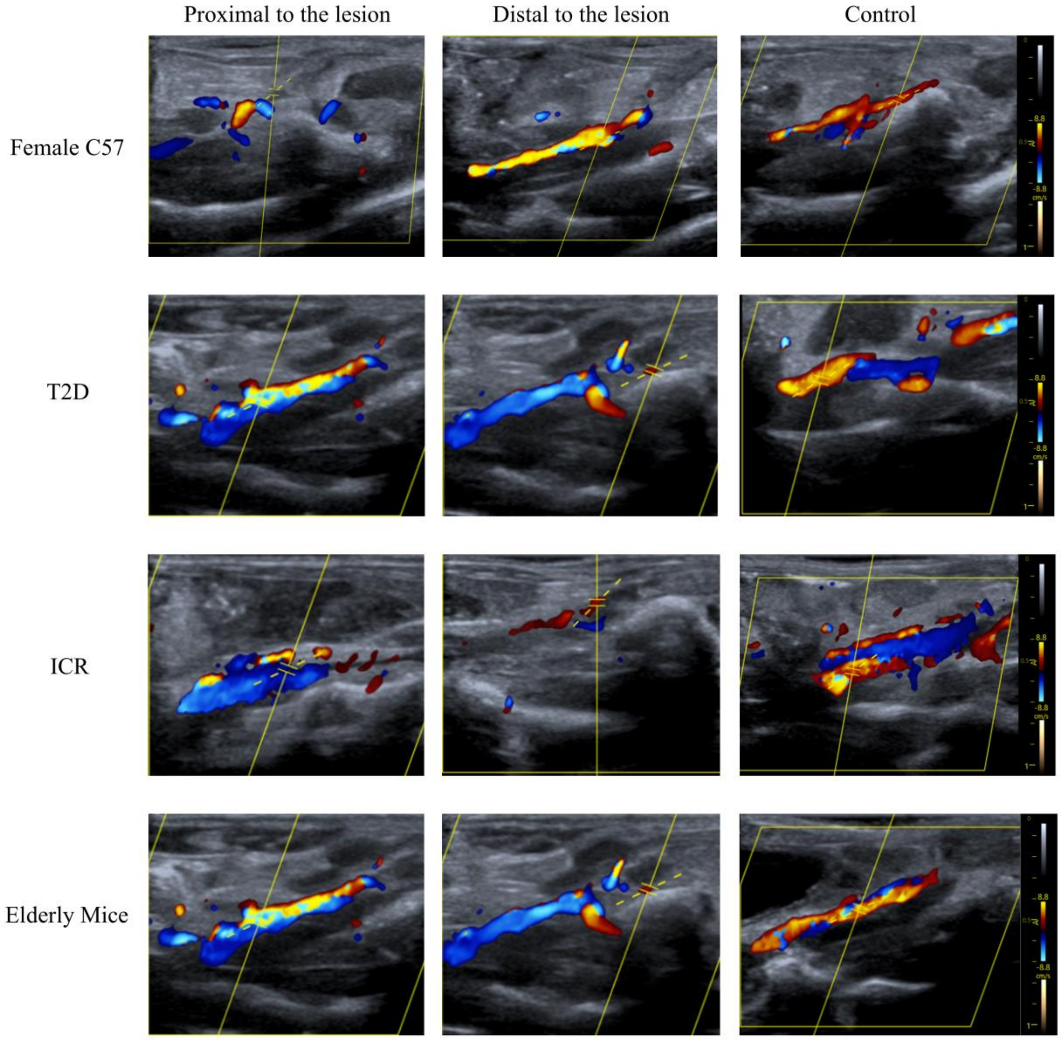

Figure S18: Ultrasound images of female C57, T2D, ICR, elderly mice after operation 3d.

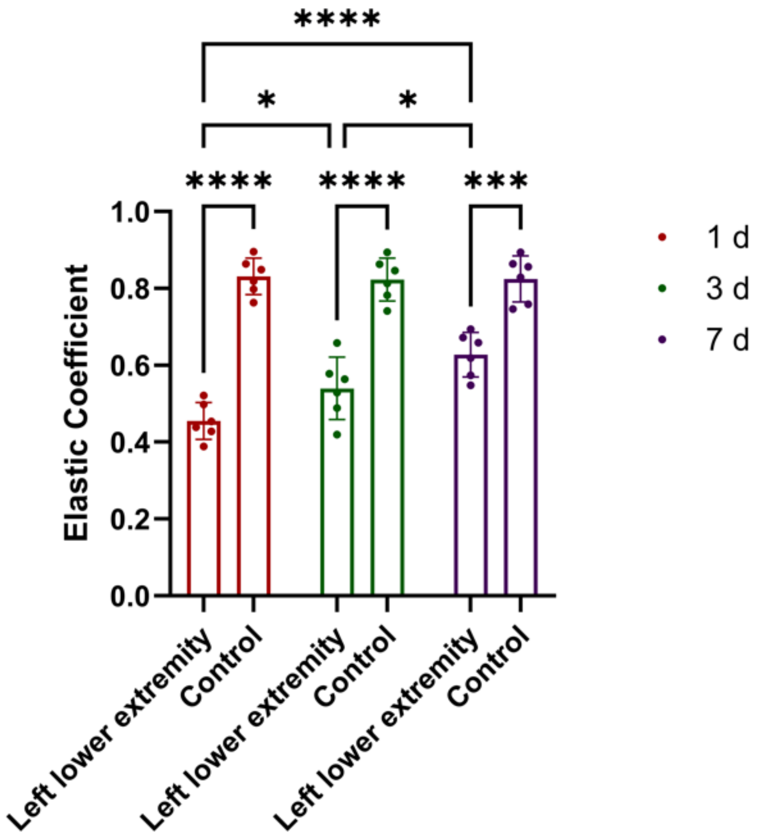

Figure S19: Elastic coefficient in ultrasound imaging after operation 1d, 3d, 7d.

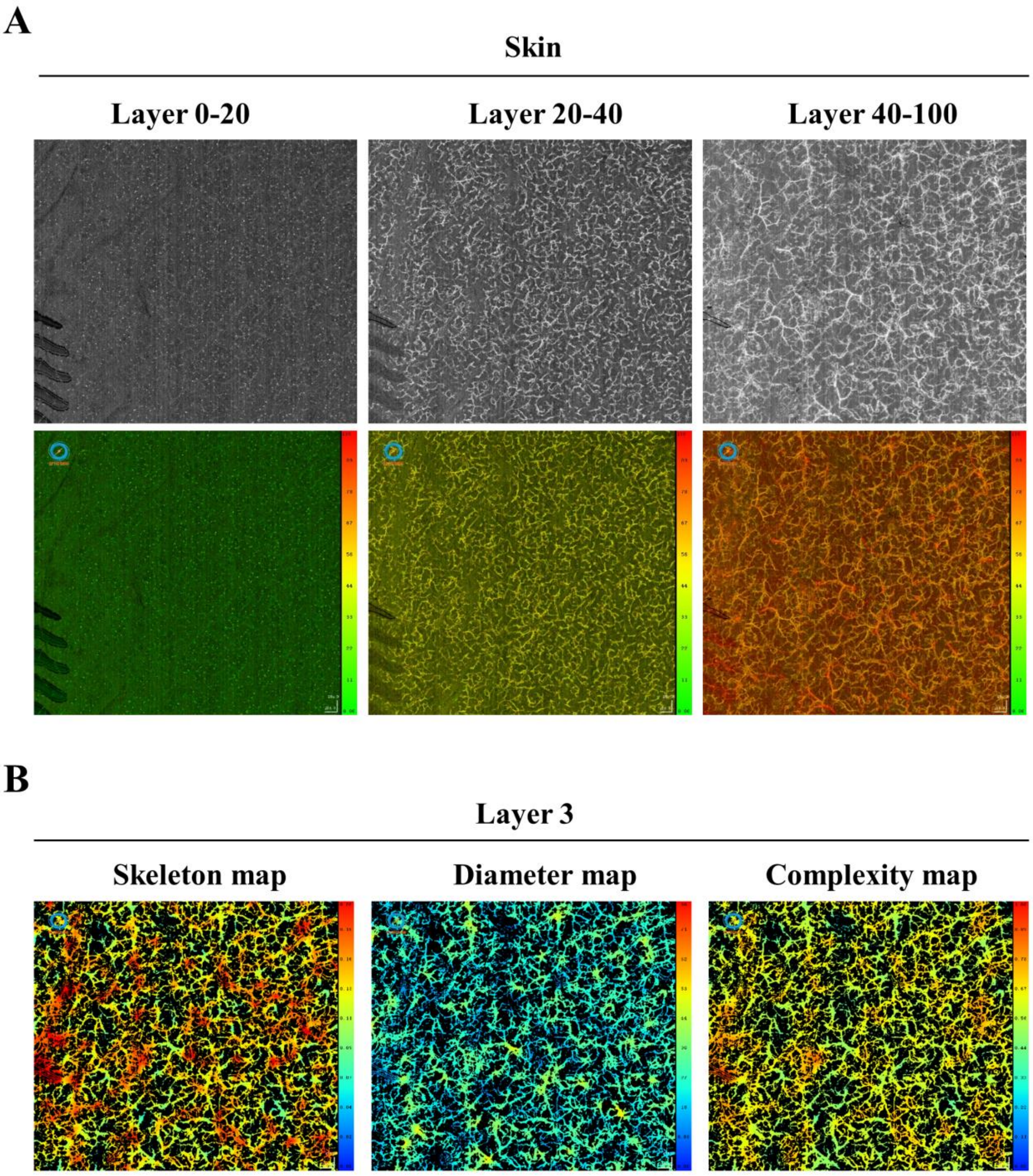

Figure S20: OCTA imaging of mouse dorsal skin. (A) OCT and OCTA imaging of mouse dorsal skin. Divided into three layers: layer 1 (0-20) , layer 2 (20-40) , layer 3 (40-100) . **(B)** Layer 3 (40-100) was selected for the following analyses: vascular skeleton map, vascular diameter map and vascular complexity map.

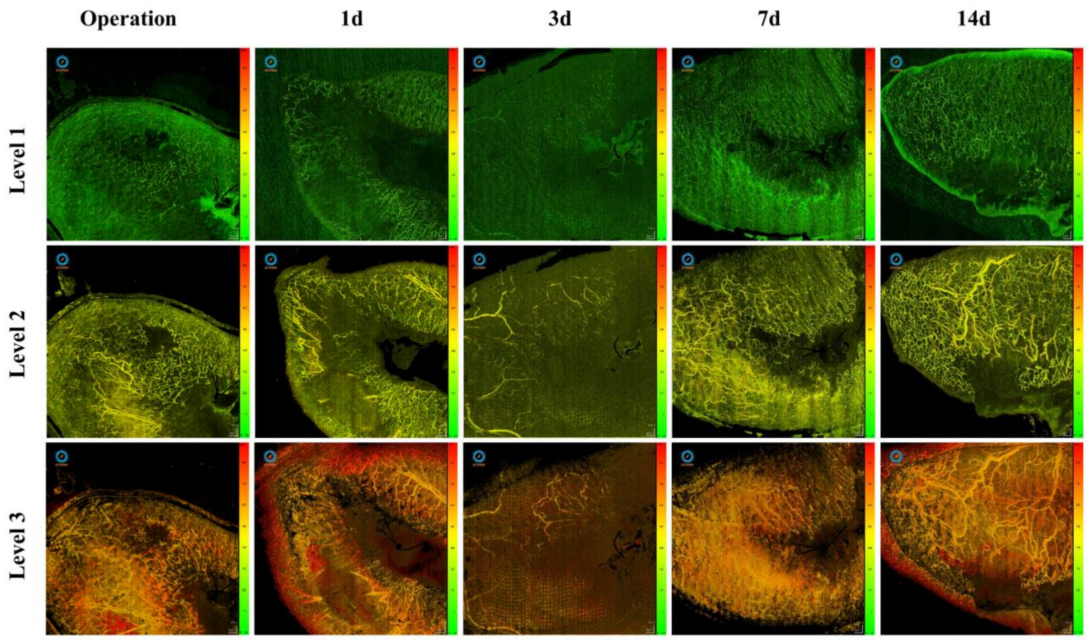

Figure S21: Continuous OCTA imaging of the damaged limbs of ALLI mice was performed.

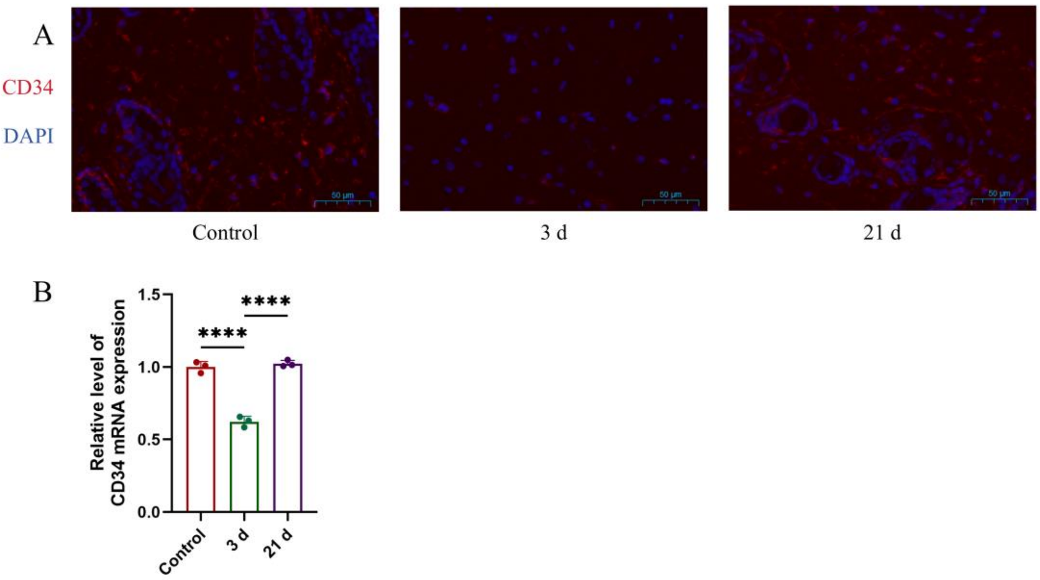

Figure S22: Immunofluorescence staining of skin of affected limb (3 and 21 days after surgery) and control group, and quantitative analysis. (A) Immunofluorescence staining. **(B)** Quantitative analysis. ****$p < 0.0001$.

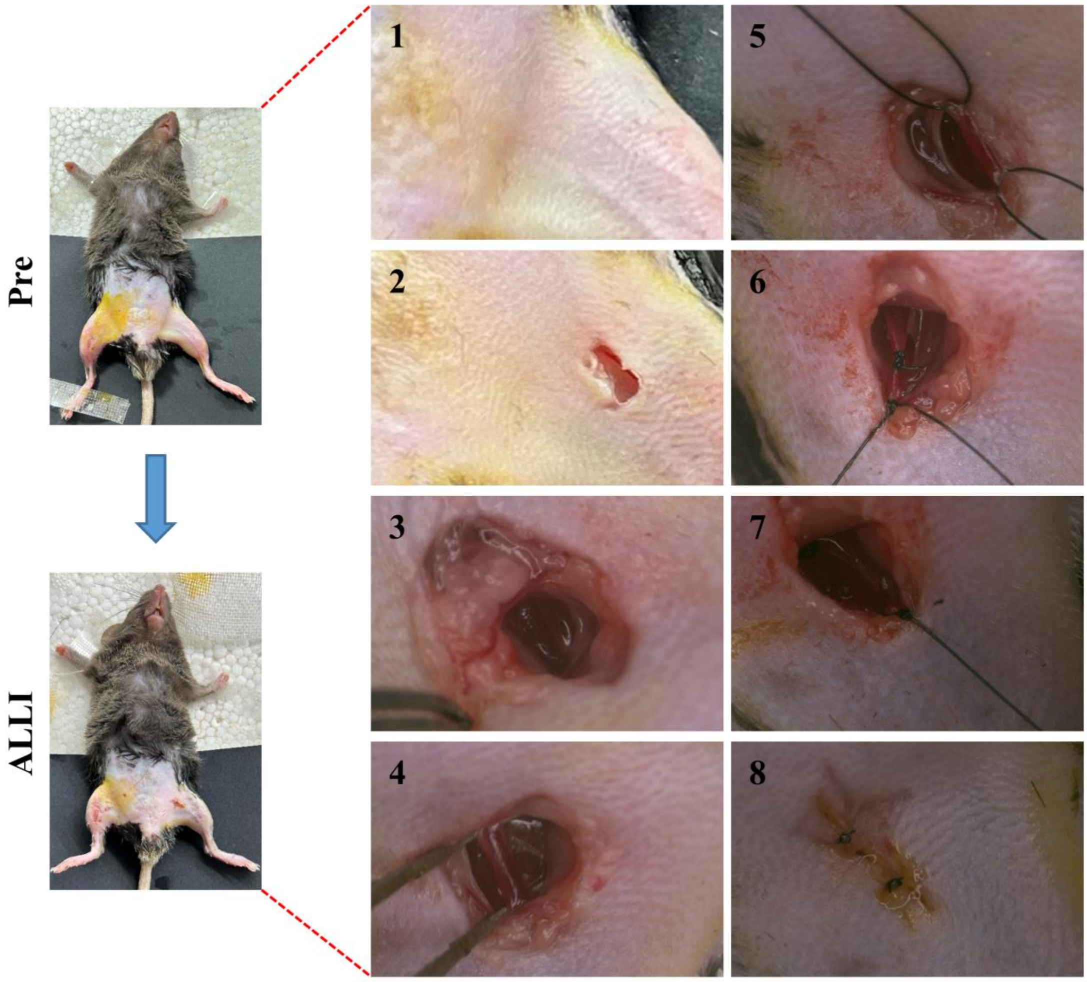

Figure S23: Procedures for ALLI models in mice.

NOTE: Please save this file locally before filling in the table, DO NOT work on the file within your internet browser as changes will not be saved. Adobe Acrobat Reader (available free here) is recommended for completion.

ARRIVE

The ARRIVE guidelines 2.0: author checklist

The ARRIVE Essential 10

These items are the basic minimum to include in a manuscript. Without this information, readers and reviewers cannot assess the reliability of the findings.

Item		Recommendation	Section/line number, or reason for not reporting
**Study design**	1	For each experiment, provide brief details of study design including:	
		a. The groups being compared, including control groups. If no control group has been used, the rationale should be stated.	Supplementary Methods/lines 3-6
		b. The experimental unit (e.g. a single animal, litter, or cage of animals).	Supplementary Methods/line 9
**Sample size**	2	a. Specify the exact number of experimental units allocated to each group, and the total number in each experiment. Also indicate the total number of animals used.	Supplementary Methods/lines 20-22
		b. Explain how the sample size was decided. Provide details of any *a priori* sample size calculation, if done.	Supplementary Methods/lines 20
**Inclusion and exclusion criteria**	3	a. Describe any criteria used for including and excluding animals (or experimental units) during the experiment, and data points during the analysis. Specify if these criteria were established *a priori*. If no criteria were set, state this explicitly.	Supplementary Methods/lines 22-25
		b. For each experimental group, report any animals, experimental units or data points not included in the analysis and explain why. If there were no exclusions, state so.	Supplementary Methods/lines 25-27
		c. For each analysis, report the exact value of *n* in each experimental group.	Supplementary Methods/lines 25-27
**Randomisation**	4	a. State whether randomisation was used to allocate experimental units to control and treatment groups. If done, provide the method used to generate the randomisation sequence.	NA
		b. Describe the strategy used to minimise potential confounders such as the order of treatments and measurements, or animal/cage location. If confounders were not controlled, state this explicitly.	Supplementary Methods/lines 27-29
**Blinding**	5	Describe who was aware of the group allocation at the different stages of the experiment (during the allocation, the conduct of the experiment, the outcome assessment, and the data analysis).	Supplementary Methods/lines 27-31
**Outcome measures**	6	a. Clearly define all outcome measures assessed (e.g. cell death, molecular markers, or behavioural changes).	Supplementary Methods/lines 25-27
		b. For hypothesis-testing studies, specify the primary outcome measure, i.e. the outcome measure that was used to determine the sample size.	NA
**Statistical methods**	7	a. Provide details of the statistical methods used for each analysis, including software used.	Materials and Methods/lines 600-608
		b. Describe any methods used to assess whether the data met the assumptions of the statistical approach, and what was done if the assumptions were not met.	Materials and Methods/lines 601-603
**Experimental animals**	8	a. Provide species-appropriate details of the animals used, including species, strain and substrain, sex, age or developmental stage, and, if relevant, weight.	Supplementary Methods/lines 3-5, 20-22
		b. Provide further relevant information on the provenance of animals, health/immune status, genetic modification status, genotype, and any previous procedures.	Supplementary Methods/lines 3-4
**Experimental procedures**	9	For each experimental group, including controls, describe the procedures in enough detail to allow others to replicate them, including:	Materials and Methods/lines 473-579
		a. What was done, how it was done and what was used.	Materials and Methods/lines 510-512, 527-529, 548-551, 577-579
		b. When and how often.	
		c. Where (including detail of any acclimatisation periods).	Supplementary Methods/lines 31-33
		d. Why (provide rationale for procedures).	Materials and Methods/lines 474-478
**Results**	10	For each experiment conducted, including independent replications, report:	Results/lines 309-318
		a. Summary/descriptive statistics for each experimental group, with a measure of variability where applicable (e.g. mean and SD, or median and range).	
		b. If applicable, the effect size with a confidence interval.	Figure 2-8

The Recommended Set

These items complement the Essential 10 and add important context to the study. Reporting the items in both sets represents best practice.

Item		Recommendation	Section/line number, or reason for not reporting
**Abstract**	11	Provide an accurate summary of the research objectives, animal species, strain and sex, key methods, principal findings, and study conclusions.	Abstract/lines 37-48
**Background**	12	a. Include sufficient scientific background to understand the rationale and context for the study, and explain the experimental approach.	Introduction/ lines 62-91
		b. Explain how the animal species and model used address the scientific objectives and, where appropriate, the relevance to human biology.	Introduction/ lines 93-108
**Objectives**	13	Clearly describe the research question, research objectives and, where appropriate, specific hypotheses being tested.	Introduction/lines 109-115
**Ethical statement**	14	Provide the name of the ethical review committee or equivalent that has approved the use of animals in this study, and any relevant licence or protocol numbers (if applicable). If ethical approval was not sought or granted, provide a justification.	lines 800-802
**Housing and husbandry**	15	Provide details of housing and husbandry conditions, including any environmental enrichment.	Supplementary lines 27-28
**Animal care and monitoring**	16	a. Describe any interventions or steps taken in the experimental protocols to reduce pain, suffering and distress.	Supplementary lines 10-19
		b. Report any expected or unexpected adverse events.	Supplementary lines 25-26
		c. Describe the humane endpoints established for the study, the signs that were monitored and the frequency of monitoring. If the study did not have humane endpoints, state this.	Supplementary lines 27-33
**Interpretation/ scientific implications**	17	a. Interpret the results, taking into account the study objectives and hypotheses, current theory and other relevant studies in the literature.	Discussion/lines 339-378
		b. Comment on the study limitations including potential sources of bias, limitations of the animal model, and imprecision associated with the results.	Discussion/lines 450-463
**Generalisability/ translation**	18	Comment on whether, and how, the findings of this study are likely to generalise to other species or experimental conditions, including any relevance to human biology (where appropriate).	Results/lines 321-337
**Protocol registration**	19	Provide a statement indicating whether a protocol (including the research question, key design features, and analysis plan) was prepared before the study, and if and where this protocol was registered.	lines 808-812
**Data access**	20	Provide a statement describing if and where study data are available.	lines 804-806
**Declaration of interests**	21	a. Declare any potential conflicts of interest, including financial and non-financial. If none exist, this should be stated.	lines 797-797
		b. List all funding sources (including grant identifier) and the role of the funder(s) in the design, analysis and reporting of the study.	lines 785-794
